# Detecting the *FLJ22447* lncRNA in Ovarian Cancer with Cyclopentane-Modified FIT-PNAs (cpFIT-PNAs)

**DOI:** 10.3390/biom14060609

**Published:** 2024-05-22

**Authors:** Sheethal Thomas Mannully, Rawan Mahajna, Huda Nazzal, Salam Maree, Hongchao Zheng, Daniel H. Appella, Reuven Reich, Eylon Yavin

**Affiliations:** 1 Institute for Drug Research, School of Pharmacy, The Hebrew University of Jerusalem, Hadassah Ein-Kerem, Jerusalem 91120, Israel; sheethal.thomas@mail.huji.ac.il (S.T.M.); rawan.mahajna1@mail.huji.ac.il (R.M.); huda.nazzal@mail.huji.ac.il (H.N.); salam.maree@mail.huji.ac.il (S.M.); reuvenr@ekmd.huji.ac.il (R.R.); 2 Synthetic Bioactive Molecules Section, Laboratory of Bioorganic Chemistry (LBC), National Institute of Diabetes and Digestive and Kidney Diseases (NIDDK), National Institutes of Health, 8 Center Drive, Room 404, Bethesda, MD 20892, USA; hongchao.zheng@nih.gov (H.Z.); appellad@niddk.nih.gov (D.H.A.)

**Keywords:** cyclopentane modified FIT-PNA, *FLJ22447* lncRNA, ovarian cancer, cancer-associated fibroblasts, cell-penetrating peptide, RNA biomarker

## Abstract

Ovarian cancer (OC) is one of the most lethal gynecologic cancers that is typically diagnosed at the very late stage of disease progression. Thus, there is an unmet need to develop diagnostic probes for early detection of OC. One approach may rely on RNA as a molecular biomarker. In this regard, *FLJ22447* lncRNA is an RNA biomarker that is over-expressed in ovarian cancer (OC) and in cancer-associated fibroblasts (CAFs). CAFs appear early on in OC as they provide a metastatic niche for OC progression. FIT-PNAs (forced intercalation-peptide nucleic acids) are DNA analogs that are designed to fluoresce upon hybridization to their complementary RNA target sequence. In recent studies, we have shown that the introduction of cyclopentane PNAs into FIT-PNAs (cpFIT-PNA) results in superior RNA sensors. Herein, we report the design and synthesis of cpFIT-PNAs for the detection of this RNA biomarker in living OC cells (OVCAR8) and in CAFs. cpFIT-PNA was compared to FIT-PNA and the cell-penetrating peptide (CPP) of choice was either a simple one (four L-lysines) or a CPP with enhanced cellular uptake (CLIP6). The combination of CLIP6 with cpFIT-PNA resulted in a superior sensing of FLJ22447 lncRNA in OVCAR8 cells as well as in CAFs. Moreover, incubation of CLIP6-cpFIT-PNA in OVCAR8 cells leads to a significant decrease (ca. 60%) in *FLJ22447* lncRNA levels and in cell viability, highlighting the potential theranostic use of such molecules.

## 1. Introduction

Ovarian cancer is the second most common malignancy in women over the age of 40, and the most fatal gynecologic cancer with five-year survival rates below 50% [1]. The low survival rates are due to the poor diagnosis of the disease, and many women are present with advanced disease (stage 3 or stage 4) at diagnosis when malignant cells have already disseminated to the peritoneum [2]. Diagnosis of ovarian cancer may be difficult due to its non-specific vague symptoms, and the lack of effective or sensitive clinical screening methods, which include transvaginal ultrasonography and serum cancer antigen.

Long non-coding RNAs (lncRNA) may offer advantages as cancer biomarkers, especially as they can be readily detected in biological fluids, in cell culture, or in tissues [3]. LncRNAs are highly diverse and actively present in virtually every aspect of cell biology, including cellular differentiation and proliferation, and many are associated with cancer progression [4]. *FLJ22447* lncRNA, referred to as lncRNA-CAF [5,6] is up-regulated in cancer-associated fibroblasts (CAFs) which have an important role in the prognosis of ovarian cancer. CAF, as a major component in the tumor microenvironment, can form robust crosstalk with cancer cells, forming a pre-metastasis niche. Hence, the detection of a CAF specific biomarker may help in early-stage detection of ovarian cancer.

PNA (peptide nucleic acid) is a DNA mimic with high binding affinity to complementary DNA/RNA [7,8]. PNA molecules have been explored as therapeutic [9,10,11,12,13,14,15] and sensing [9,10,12,13,15,16] molecules.

Specifically, FIT-PNA (forced intercalation-peptide nucleic acid), originally developed by the Seitz group [17,18], is a highly sensitive RNA/DNA sensor. FIT-PNAs are “light-up” probes that turn on their fluorescence upon hybridization with their complementary RNA target. In the FIT-PNA design, a surrogate base that is a mono-methine cyanine dye (e.g., TO, JO, BisQ) replaces one of the natural PNA bases (typically a purine). In duplex form (FIT-PNA: RNA), the increased rigidity surrounding the cyanine dye results in the above-mentioned light-up property of these molecules [19]. Several reports have shown the diagnostic potential of such RNA sensors in cancer [20,21,22,23,24], and in a variety of infectious diseases [25,26,27,28,29,30].

Previously, we have shown that rigidifying the local environment of the surrogate base (BisQ) in a model FIT-PNA by introducing a cyclopentane-modified PNA monomer/s [31,32] adjacent to BisQ results in a substantial increase in the molecule’s brightness and quantum yield [33]. This strategy was recently shown to be translated for the detection of an mRNA in *Plasmodium falciparum* that is associated with drug resistance to the antimalarial drug, artemisinin [34]. 

A variety of cell-penetrating peptides (CPPs) have been used as a simple strategy for PNA delivery into cells including several recent in vitro [35,36,37,38,39,40,41,42,43,44] and in vivo [45,46] studies in eukaryotes as well as bacteria [47,48,49,50,51,52,53,54,55]. As PNA is synthesized by standard peptide chemistry (Fmoc or t-BOC), the introduction of a CPP to either C or N termini is straightforward. In a previous study, we have reported on the splice switching activity of PNA [56]. In this report, we have used a CPP termed CLIP6 (intrinsically disordered peptide [57]) that employs non-endosomal mechanisms to cross cellular membranes. Herein, we have combined CLIP6 and cpFIT-PNA as an approach to develop a superior RNA sensor for the detection of the OC biomarker, *FLJ22447* lncRNA. 

## 2. Materials and Methods

### 2.1. Chemical Synthesis of FIT-PNAs and cpFIT-PNAs

#### 2.1.1. General Procedures and Materials

Manual solid-phase synthesis was performed in 5 mL polyethylene syringe reactors (Phenomenex, Torrance, CA, USA) that are equipped with a fritted disk. HPLC purifications and analysis were performed on a Dionex UltiMate 3000 HPLC system (ThermoFisher Scientific, Waltham, MA, USA) using a semi-preparative C18 reversed-phase column (Jupiter C18, 5 μm, 300 Å, 250 × 10 mm, Phenomenex, Torrance, USA). Eluents A (0.1% TFA in water) and B (Acetonitrile) were used in a linear gradient with a flow rate of 4 mL/min. Mass analysis of FIT-PNAs were detected by a Xevo Cronos mass spectrometer (Waters) in positive mode using electrospray ionization (ESI) full scan acquisition (300–1500 *m*/*z*). The capillary voltage was set to 1 kV. The desolvation temperature and desolvation gas flow were set to 500 °C and 800 L/h, respectively. The source temperature was set at 150 °C. The cone voltage and the cone gas flow were set to 35 V and 50 L/h, respectively. Data acquisition and processing were carried out using MassLynx 4.2 SCN 1045 MS software (Waters, Wilmslow, Chesire, UK).

RNA oligos were purchased from IDT (Coralville, IA, USA). Fmoc/Bhoc protected PNA monomers from PolyOrg Inc. (Leominster, MA, USA). Fmoc-protected amino acids and reagents for solid-phase synthesis were purchased from Merck (Darmstadt, Germany), Chem-Impex (Wood Dale, IL, USA), and Bio-Lab LTD (Jerusalem, Israel). BisQ monomer was synthesized as previously reported. [23]

#### 2.1.2. Solid-Phase Synthesis of FIT-PNA

##### Coupling of First Amino Acid (L-Lysine) onto NovaSyn TGA Resin

The resin (250 mg, 0.2 mmol/g) was allowed to swell in 10 mL DMF for 30 min. For pre-activation, DIC (5 eq.) and DMAP (0.1 eq.) were added to a solution of Fmoc-L-Lysine(t-BOC)-OH (10 eq.) in DCM (15 mL) in an ice bath. After 15 min, the mixture was evaporated, re-dissolved in dry DMF, and added to the resin. After 2.5 h, the resin was washed with DMF (5 × 2 mL) and DCM (5 × 2 mL) and the procedure was repeated.

- Fmoc Cleavage. A solution of DMF/piperidine (4:1, 1 mL) was added to the resin. After 2 min, the procedure was repeated. Finally, the resin was washed with DMF (3 × 1 mL) and DCM (3 × 1 mL).
- Coupling of Fmoc-Bhoc-PNA-Monomers. An amount of 4 eq. of PNA monomer, 4 eq. HATU, 4 eq. HOBt, and 8 eq. of dry DIPEA in DMF (to 0.1 M PNA) were mixed in a glass vial equipped with a screw cap. After 3 min of pre-activation, the solution was transferred to the resin. After 60 min, the reaction mixture was discarded, and the resin was washed with DMF (2 × 1 mL) and DCM (2 × 1 mL).
- Coupling of BisQ. An amount of 4 eq. of BisQ monomer, 4 eq. HATU, 4 eq. HOBt, and 8 eq. of dry DIPEA in DMF (to 0.1 M BisQ monomer) were mixed in a glass vial equipped with screw cap. Following 3 min of pre-activation, the solution was transferred to the resin. After 60 min, the procedure was repeated, and finally, the resin was washed with DMF (2 × 1 mL) and DCM (2 × 1 mL).
- Cleavage of PNA from resin. An amount of 1 ml TFA was added to the dry resin. After 2 h, another portion of TFA was added. The combined TFA solutions were concentrated in vacuo.
- PNA Purification. PNAs were precipitated from the concentrated TFA solution by addition of cold diethyl ether (10 mL). The precipitate was collected by centrifugation and decantation of the supernatant. The residue was dissolved in water and purified by semi-preparative HPLC. The purified PNAs were analyzed by ESI-MS. In some FIT-PNAs (e.g., K4 FIT-PNA, Table 1) a difference of 5 Daltons is found between observed and predicted masses, which is in the range of the MS accuracy (below 0.1%).

### 2.2. In Vitro Studies with Synthetic RNA

#### 2.2.1. Tm Measurements

Melting temperatures (Tm) of the PNA/RNA duplexes were estimated from UV melting curves measured on an Evolution One Plus UV-Vis Spectrophotometer (Thermo Fisher Scientific, Waltham, USA). Solutions of the FIT-PNAs/cpFIT-PNAs and their complementary RNAs (1:1 ratio) were prepared in PBS buffer (pH 7.0) and adjusted to a final duplex concentration of 2 µM. Prior to analysis, the samples were heated from 20 °C to 90 °C at a rate of 5 °C/min and then cooled to the starting temperature at a rate of 2 °C/min. The change in absorbance was monitored at 260 nm by increasing the temperature to 90 °C at a rate of 1 °C/min. 

#### 2.2.2. Fluorescence Measurements

Fluorescence spectra of FIT-PNAs with/out synthetic RNA was measured using a Jasco FP-6500 Spectrofluorometer (Jasco Inc. co., LTD, Tokyo, Japan) with a slit width set to 1 nm and a response rate of 0.5 s. Excitation was set to 580 nm and the emission spectra were recorded from 590 nm to 800 nm. Solutions of cpFIT-PNA/FIT-PNAs (1 μM) and synthetic RNA (2 μM) were prepared in 1× PBS. The hybridization of cpFIT-PNA/FIT-PNAs with synthetic RNA was performed by incubation at 37 °C for 3 h at a ratio of 0.5:1. The RNA sequence used was 5′-AAGAUCUAACUGUC. 

### 2.3. Cell Lines and Culture Conditions 

Ovarian cancer cell line OVCAR8 and MRC5 were purchased from American Type Culture Collection (ATCC) and maintained according to the guidelines provided by the repository. Normal fibroblast (NF) and cancer-associated fibroblast (CAF) primary cells were isolated from human omentum tissues taken during OC surgery, generously provided by Dr. Tamar Perri (Hadassah Medical Centre, Jerusalem, Israel). OVCAR8 cells were cultured in RPMI 1640 medium (ThermoFisher Scientific, Waltham, USA) supplemented with 5% fetal bovine serum (FBS) (Sigma-Aldrich, St. Louis, MO, USA). MRC5 cells were grown in Dulbecco’s Modified Eagle Medium (DMEM) (Sigma Aldrich, St. Louis, MO, USA) with 10% FBS. NF and CAF primary cells were grown in specially prepared basal medium containing MEM and Ham’s Nutrient Mixture F12 (Biological Industries, Beit-Haemek, Israel), Medium 199 (ThermoFisher Scientific, Waltham, USA), supplemented with epidermal growth factor (EGF) (ThemoFisher Scientific, Waltham, USA) and hydrocortisone (STEMCELL Technologies, Petach-Tiqva, Israel). All the media were supplemented with 1% of 100 U/mL penicillin–streptomycin (ThermoFisher Scientific, Waltham, USA), L-glutamine, sodium pyruvate, vitamins, and MEM non-essential amino acids (Biological Industries, Beit-Haemek, Israel). The cell culture was maintained at 37 °C with 5% CO_2_.

### 2.4. RT-qPCR 

Total RNA from cells were isolated using TRIzol reagent (ThermoFisher Scientific, Waltham, USA) according to manufacturer’s instructions and quantified using NanoDrop 2000 Spectrophotometer (ThermoFisher Scientific, Waltham, USA). Reverse transcription of RNA (1 μg) into cDNA was performed using QScript cDNA synthesis kit (Quantabio, Beverly, MA, USA) following manufacturer’s instructions. RT-qPCR was performed to examine the mRNA expression of FLJ22447 lncRNA, αSMA, and HIF-1α in CFX Connect Real-Time PCR Detection System (BioRad, Hercules, CA, USA) using PerfeCTa SYBER^®^ Green FastMix qPCR reagent (Quantabio, Beverly, MA, USA). The primer sequences used in this study were obtained from IDT (Coralville, USA) and HyLabs (Rehovot, Israel) and its characteristics were listed in Supplementary Table S3. The target genes were amplified using the following thermocycling conditions: initial denaturation at 95 °C for 5 min, followed by 40 cycles of 95 °C for 10 s and 60 °C for 30 s. The specificity of PCR products was analyzed by appropriate melting curves. The ratio of relative expression of target genes were calculated using 2^−ΔΔCT^ method and was normalized to the expression of housekeeping gene Glyceraldehyde 3-phosphate dehydrogenase (*GAPDH)* and Ribosomal protein lateral stalk subunit P0 (*RPLP0*).

### 2.5. Confocal Microscopy Analysis 

OVCAR8 cells (40 × 10^3^) were plated on µ slide 8 well (ibidi GmbH, Gräfelfing, Germany) and cultured at 37 °C, 5% CO_2_ for 24 h. The cells were washed with 1× PBS and incubated with 2 µM FIT-PNAs in RPMI with 5% FBS medium for 3 h at 37 °C. After incubation, the cells were washed twice with 1× PBS followed by nuclear staining using Hoechst (1 µg/mL) for 15 min at 37 °C. The cells were washed twice with 1× PBS followed by addition of 300 µL of 1× PBS into wells for the observation of living cells. OVCAR8 cells not treated with FIT-PNA was used as the control for the experiment. The cells were observed with Nikon AIR+ confocal microscope using 20× Plan-flour objective N.A (Core Research Facilities, The Hebrew University of Jerusalem, Jerusalem, Israel) and the images were processed using NIS-Elements AR software (version 4.40). The imaging conditions such as laser intensities, photomultiplier gain and offset, confocal aperture was kept constant throughout the observation of different FIT-PNAs so that the fluorescence intensities analyzed represented the correct difference in the cellular uptake of different FIT-PNAs. The focal plane for each image was selected depending upon the highest intensity of mCherry fluorescence by FIT-PNA. 

### 2.6. Flow Cytometry Analysis 

The FACS analysis of FIT-PNA uptake was performed by growing OVCAR8 (150 × 10^3^) and CAF primary cells (150 × 10^3^) to approximately 80% confluency in 6-well plates. After two washes with 1× PBS, the cells were incubated with 2 µM different FIT-PNAs for 3 h at 37 °C. After thorough washing, the cells were harvested using 0.25% Trypsin-EDTA, centrifuged at 1500 rpm and resuspended in 300 µL 1× PBS which was further filtered, and analyzed using Fortessa FACS analyzer (Core Research Facilities, The Hebrew University of Jerusalem, Jerusalem, Israel). The cells were gated according to the positivity of mCherry detected in those cells treated with FIT-PNAs in comparison to its untreated control cells. A minimum of 10,000 events under gating, defined on a forward and side scattered plots, were acquired per sample. The results were analyzed using FlowJo 10.10 software and the histogram was plotted at logarithmic scale.

### 2.7. MTT Assay

For the cell cytotoxicity assays, OVCAR8 cells were seeded to 24-well plates at the density of 1 × 10^6^ cells/well and 6 × 10^4^ cells/well. After 24 h, cell culture plate containing initial seeding of 1 × 10^6^ cells/well and 6 × 10^4^ cells/well were treated with 2 μM of cpFIT-PNAs in RPMI medium for 3 h and 48 h, respectively. After the treatment, the medium was removed and replaced with 200 μL of fresh RPMI medium containing 0.5 mg/mL of 3-(4,5-dimethyltiazol-2-yl)-2,5-diphenyltetrazolium bromide (MTT) (Sigma-Aldrich, St. Louis, MO, USA). Cells were incubated for 30 min with MTT, followed by the addition of 50 μL of DMSO to each well to dissolve formazan crystals. Absorbance was measured at 570 nm wavelength using microplate reader (BMG LABTECH’s FLUOstar Omega, Ortenberg, Germany). OVCAR8 cells without cpFIT-PNA treatment were kept as control and the cell cytotoxicity was measured by the percentage of conversion of the MTT dye to purple formazan after treatment with cpFIT-PNA in comparison to non-treated cells.

### 2.8. Statistical Analysis

The statistical analysis was performed using Student’s *t*-test. All data were represented as mean ± SD assuming the data follow a normal distribution. The probability of P value less than 0.05 was considered as statistically significant. mRNA expression measured by RT-qPCR was defined relative to its expression in control cells in each experiment where each value is the average of two biological replicates with its respective duplicates.

## 3. Results

### 3.1. FIT-PNA and cpFIT-PNA Design and Synthesis

Cyclopentane modified PNAs, developed at the Appella lab [29,30] are PNA monomers with a cyclopentane backbone. These cpPNAs have been shown to increase affinities of cpPNAs to complementary DNA and are promising antisense molecules [31,32]. In a recent study [33], we have shown that introducing cpPNA adjacent to the surrogate base (BisQ) in a model 11-mer FIT-PNA results in an impressive increase in quantum yield and brightness of these RNA sensors.

**Scheme 1:**
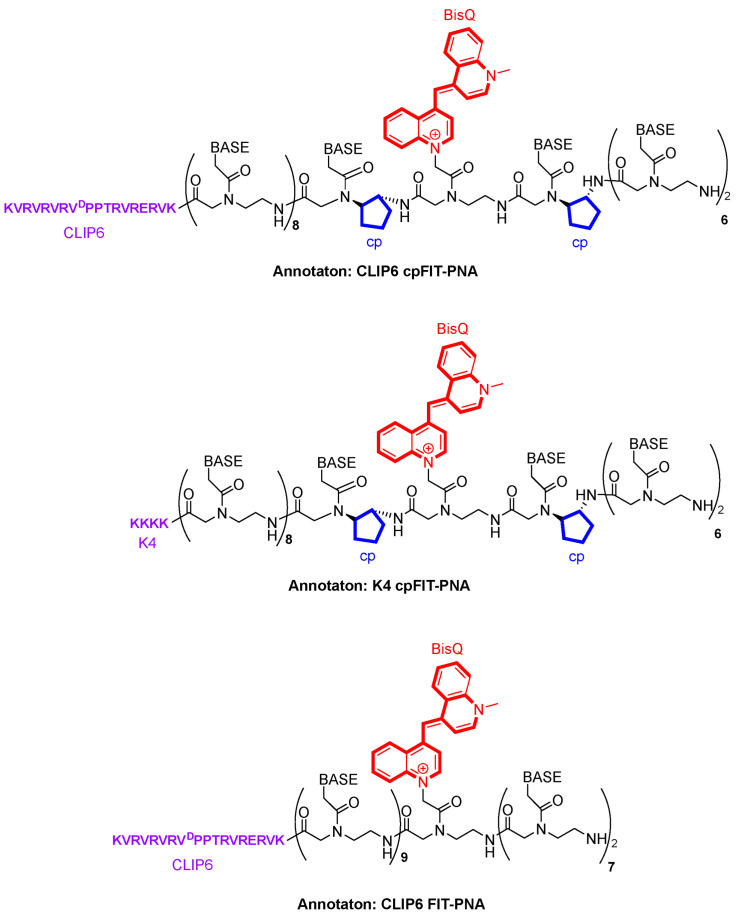
The general design of FIT-PNAs and cpFIT-PNAs.

Based on these findings, we have synthesized cpFIT-PNAs for the detection of FLJ22447 lncRNA (Scheme 1 and Table 1). To provide water solubility and cellular uptake, we introduced two different cell-penetrating peptides (CPPs) to the C-terminus of FIT-PNAs. One CPP is a very simple one that is based on a stretch of 4 Lysines (K4). The other CPP is an 18-mer peptide termed CLIP6 [57] that we have previously conjugated to splice switching PNAs [56]. 

CLIP6-PNAs were shown to have superior cellular uptake in cancer cells (U87-GBM, glioblastoma multiform) [55]. The FIT-PNA and cpFIT-PNA sequence is a 17-mer PNA that was selected based on the accessibility of this probe to an unfolded region in FLJ22447 lncRNA, as predicted by the RNA-fold software (http://rna.tbi.univie.ac.at/cgi-bin/RNAWebSuite/RNAfold.cgi (accessed on 4 March 2024). In addition, the PNA sequence was verified as specific to this lncRNA based on RNA blast analysis. A scrambled cpFIT-PNA sequence was also designed for the K4 series (Table 1). This scrambled FIT-PNA has no more than 82% similarity to RNA transcripts in the human genome as verified by NCBI blast. The general chemical structures are shown in Scheme 1. 

As shown in Scheme 1, the cpPNA monomers are placed adjacent to the BisQ monomer (e.g., cpT-BisQ-cpA). In this design, we define such molecules as cpFIT-PNA. 

### 3.2. Flourescence Enhancements and Binding Affinities of FIT-PNAs and cpFIT-PNA with Synthetic RNA

**Figure 1:**
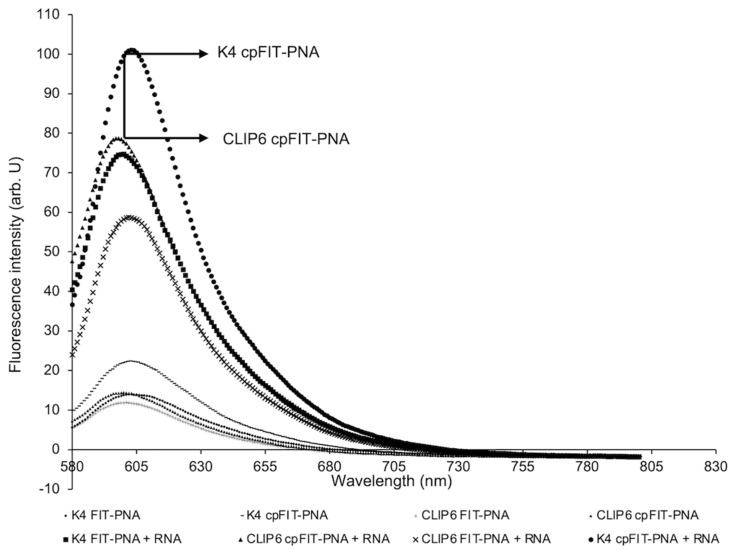
Fluorescence emission spectrum of FIT-PNAs and cpFIT-PNAs towards synthetic RNA. (λex = 580 nm; λem = 590 nm). [FIT-PNA] = 0.5 μM and [RNA] = 1 μM.

Fluorescence of FIT-PNAs/cpFIT-PNAs with synthetic RNA was measured (Figure 1) and, as expected, the cyclopentane modified FIT-PNAs (cpFIT-PNAs) exhibited higher fluorescence in duplex form, in comparison to their unmodified counterparts (FIT-PNA). Interestingly, the CLIP6 CPP leads to quenching of fluorescence in the duplex form (highlighted in Figure 1). Next, we measured the binding affinities of FIT-PNAs/cpFIT-PNAs with synthetic RNA by measuring the melting curves of these duplexes (Scheme 1 and Table 1). There is a significant difference in Tm values (ca. ΔTm = 8 °C) between CLIP6 and K4 conjugated FIT-PNAs/cpFIT-PNAs.

This may suggest that the CLIP6 CPP interacts with the PNA-RNA duplex leading to higher stability of the duplex and, at the same time, to fluorescence quenching. As CLIP6 has multiple positive charges (9 in total), it is reasonable to suggest that this peptide interacts with the negatively charged PNA–RNA duplex via the negatively charged phosphate backbone of the RNA strand. In addition, a ΔTm = 3 °C and 3.3 °C is found for cpFIT-PNAs (in comparison to FIT-PNAs), as previously shown. 

### 3.3. FIT-PNAs and cpFIT-PNAs Detect FLJ22447 lncRNA in Living OVCAR8 Cells and CAFs

#### 3.3.1. Cellular Uptake of FIT-PNAs and cpFIT-PNA in OVCAR8 Cells as Corroborated by Confocal Microscopy

**Figure 2:**
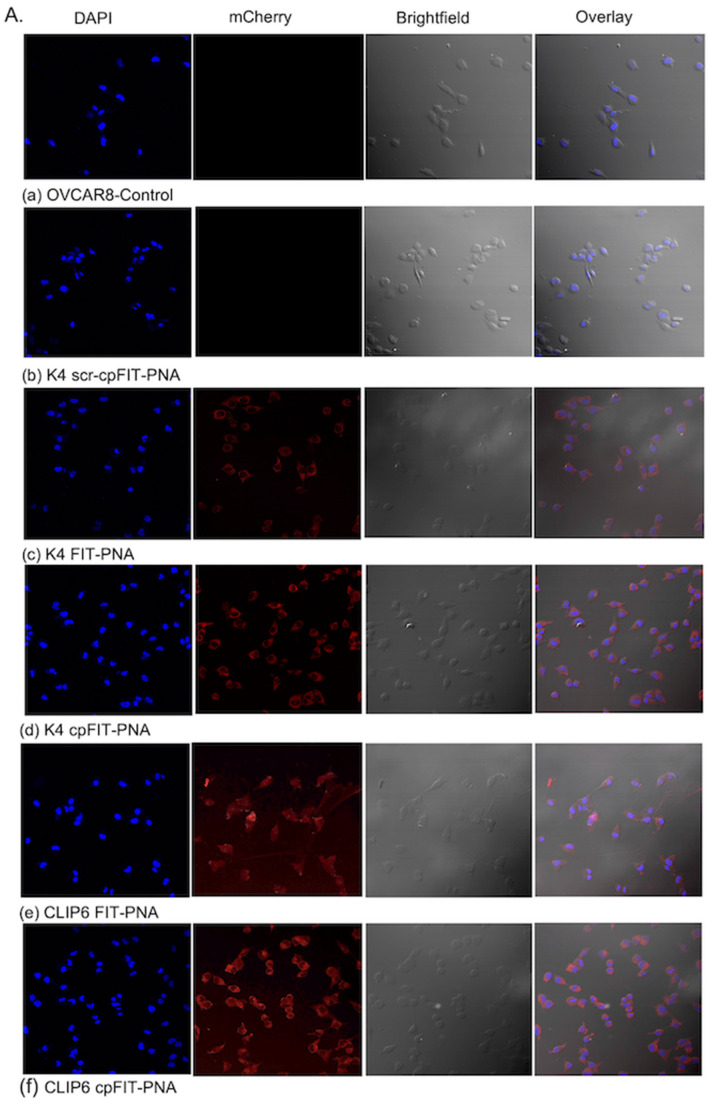

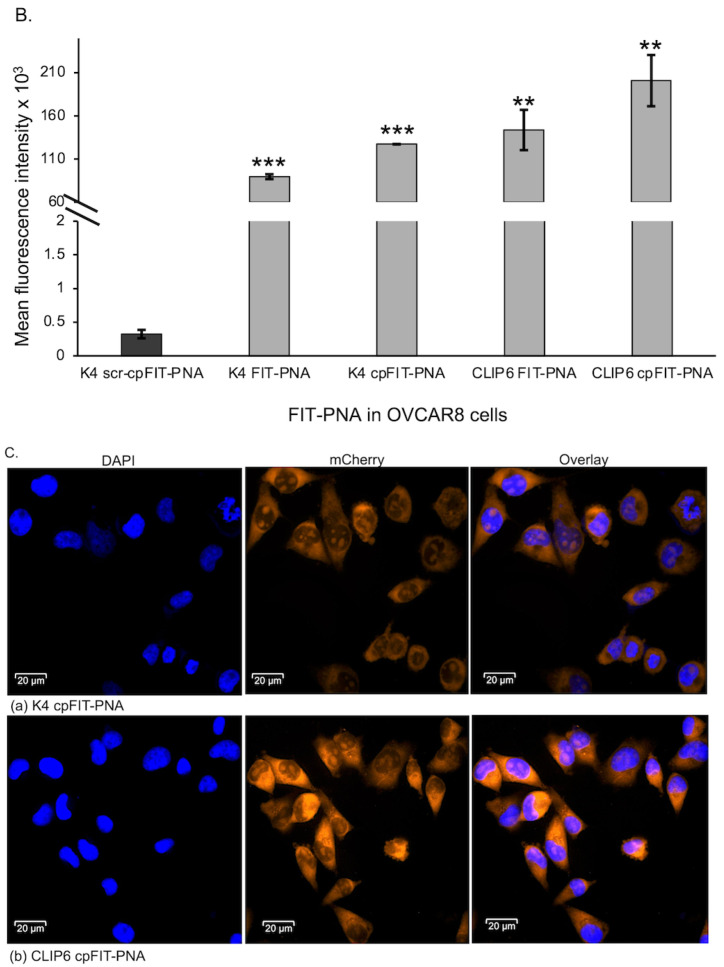
cpPNA and CLIP6 enhance fluorescence of FIT-PNAs in OVCAR8 living cells. (**A**) OVCAR8 cells treated with 2 μM FIT-PNA/cpFIT-PNA for 3 h at 37 °C were washed twice with 1× PBS, followed by nuclei staining with Hoechst stain (1 μg/mL) and washing twice with 1× PBS. Confocal imaging at 20 × magnification. (**a**) OVCAR8 cells as control; OVCAR8 cells treated with (**b**) K4 scr-cpFIT-PNA; (**c**) K4 FIT-PNA; (**d**) K4 cpFIT-PNA; (**e**) CLIP6 FIT-PNA; (**f**) CLIP6 cpFIT-PNA, respectively. (**B**) Quantification of the mean fluorescence intensity signal of OVCAR8 cells treated with 2 μM FIT-PNA/cpFIT-PNA using ImageJ 1.53t software. Statistical analysis performed using Student’s *t*-test; error bars represent mean ± SEM; ** *p* < 0.01, *** *p* < 0.001. (**C**) Magnified images (40×) for cellular uptake for K4 cpFIT-PNA and CLIP6 cpFIT-PNA in OVCAR8 cells.

Cellular uptake of FIT-PNAs and cpFIT-PNAs in OVCAR8 cells was analyzed using live cell confocal microscopy (Figure 2A). Cellular uptake of FIT-PNAs and cpFIT-PNAs was evident in target cells after 3 h incubation (Figure 2(Ac–Af)). Two different CPPs were compared: a simple CPP composed of 4 lysine peptide (K4) and an 18-mer CPP- CLIP6 [57]. Both CPPs were conjugated at the C terminus of the FIT-PNA targeting *FLJ22447* lncRNA (Table 1). The various FIT-PNAs and cpFIT-PNAs showed differences in cellular uptake in OVCAR8 cells in terms of fluorescence intensity. Further, the direct visualization of cells incubated with different FIT-PNAs and cpFIT-PNAs showed the *FLJ22447* lncRNA targeted K4 and CLIP6-FIT-PNAs localized predominantly in the cytoplasm (Figure 2C). Most importantly, the combination of cpFIT-PNA and CLIP6 (Figure 2(Af)) showed remarkable increase in its mean fluorescence intensity (Figure 2B) in comparison to its corresponding FIT-PNA without cyclopentane and CLIP6 (K4 FIT-PNA) (Figure 2(Ac),B). 

#### 3.3.2. Cellular Uptake of FIT-PNAs and cpFIT-PNA in OVCAR8 Cells and CAFs as Corroborated by FACS Analysis

**Figure 3:**
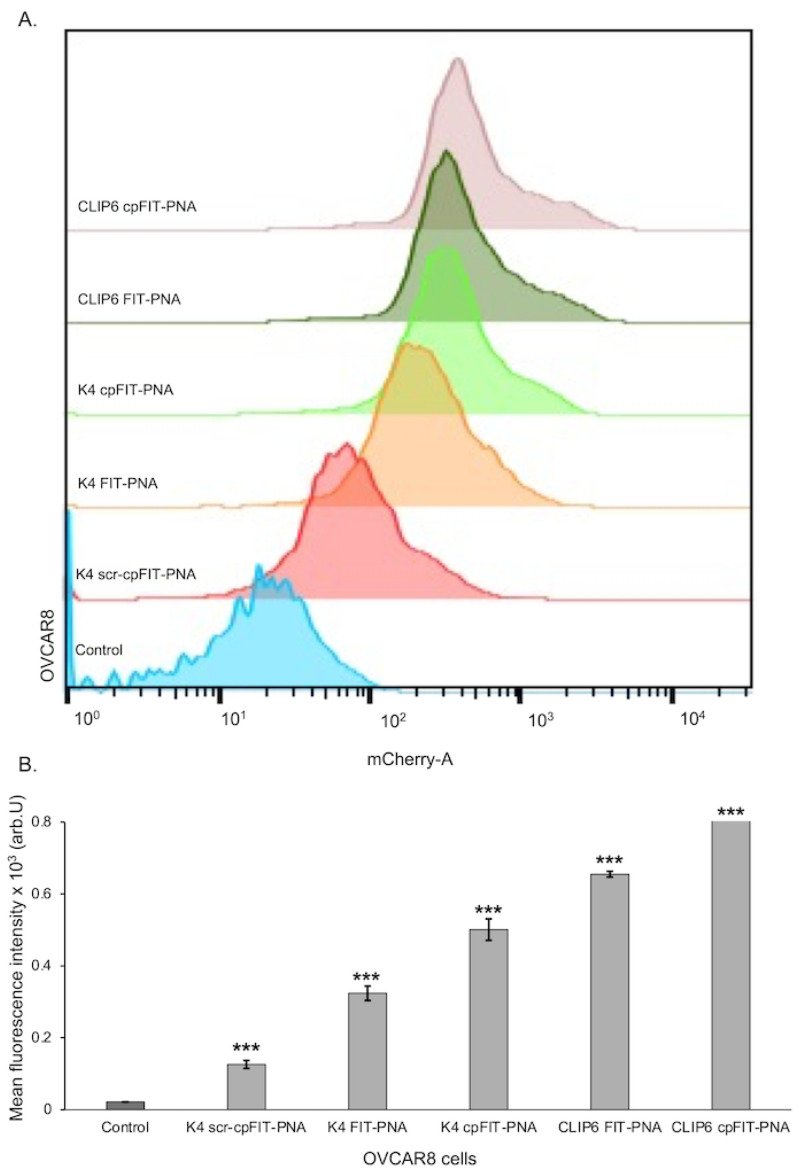
FIT-PNA/cpFIT-PNA uptake into OC cells (OVCAR8) as analyzed by flow cytometry. (**A**) Histogram of flow cytometry analysis in OVCAR8 cells treated with 2 μM FIT-PNA/cpFIT-PNA for 3 h at 37 °C. Histogram illustrates the mean fluorescence intensity plotted in horizontal axis against the number of cell events detected in the vertical axis. Both cp and CLIP6 enhance fluorescence of FIT-PNAs in OVCAR8 cells. (**B**) Mean fluorescence intensity of FIT-PNA/cpFIT-PNA in OVCAR8 cells. Statistical analysis performed using FlowJo 10.10 software and Student’s *t*-test; error bars represent mean ± SEM; *** *p* < 0.001.

**Figure 4:**
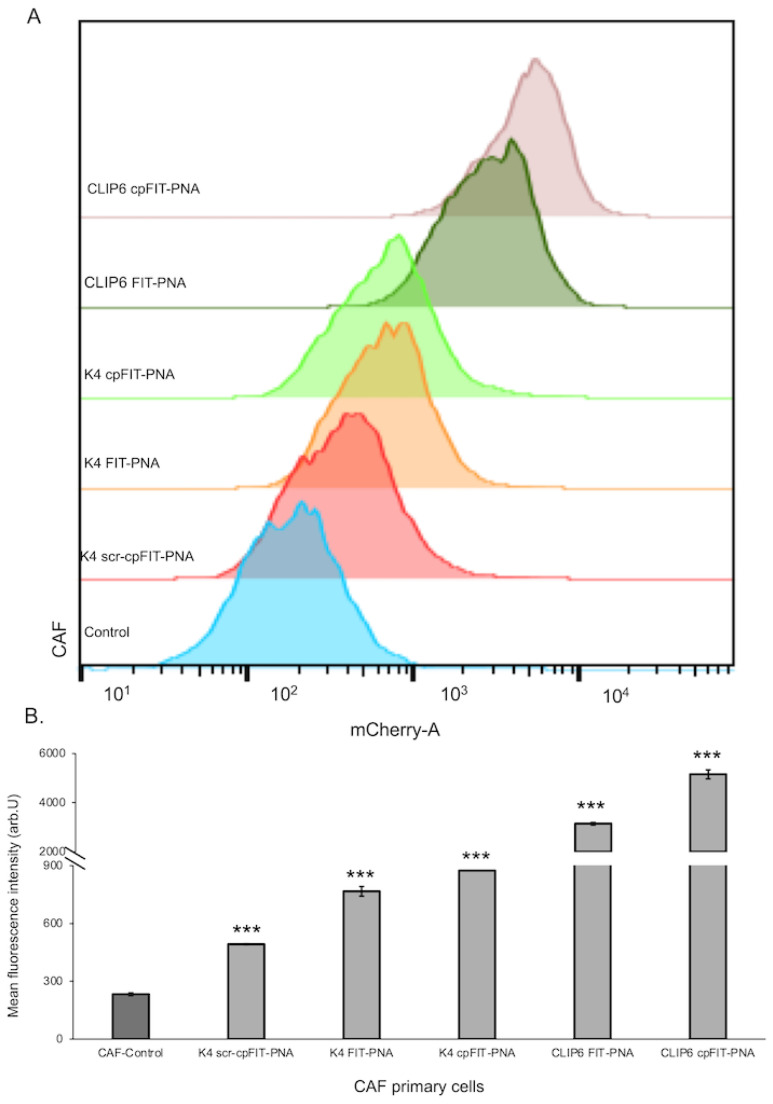
FIT-PNA/cpFIT-PNA uptake into CAF primary cells as analyzed by flow cytometry. (**A**) Histogram of flow cytometry analysis in CAF primary cells treated with 2 μM FIT-PNA/cpFIT-PNA for 3 h at 37 °C. Histogram illustrates the mean fluorescence intensity plotted in horizontal axis against the number of cell events detected in the vertical axis. Both cp and CLIP6 enhance fluorescence of FIT-PNAs in CAF primary cells. (**B**) Mean fluorescence intensity of FIT-PNA/cpFIT-PNA in CAF primary cells. Statistical analysis performed using FlowJo 10.10 software and Student’s *t*-test; error bars represent mean ± SEM; *** *p* < 0.001.

The cellular uptake of FIT-PNAs and cpFIT-PNAs in cells was further characterized by FACS analysis. The introduction of CPPs K4 and CLIP6 as well as cyclopentane in the FIT-PNA (cpFIT-PNA) showed an increase in fluorescence shifted towards the right position in the histogram for both OVCAR8 (Figure 3A) and CAF primary cells (Figure 4A). Further, the mean fluorescence intensity of CLIP6 cpFIT-PNA was found to be greater than all other FIT-PNAs in both OVCAR8 (Figure 3B) and CAF primary cells (Figure 4B). 

#### 3.3.3. *FLJ22447* lncRNA Expression in OVCAR8 Cells and CAFs as Corroborated by RT-qPCR

**Figure 5:**
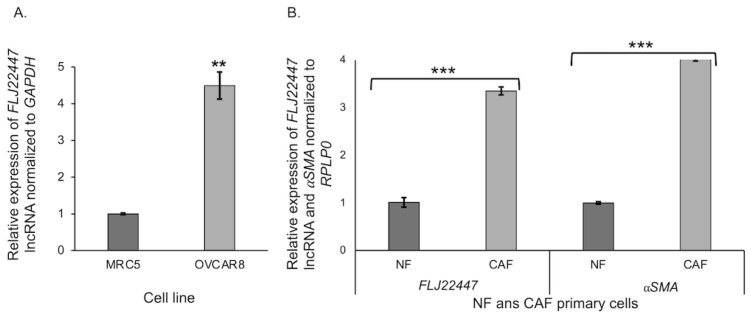
(**A**) RT-qPCR analysis of *FLJ22447* lncRNA expression in normal fibroblast (MRC5) and ovarian cancer cell OVCAR8. (**B**) RT-qPCR analysis of FLJ22447 lncRNA expression in normal fibroblast (NF) and CAF primary cells. Statistical analysis performed using Student’s *t*-test; error bars represent mean ± SEM; ** *p* < 0.01, *** *p* < 0.001.

The relative expression of *FLJ22447* lncRNA in OVCAR8 and CAF primary cells in comparison to normal fibroblast was analyzed using RT-qPCR (Figure 5). *FLJ22447* lncRNA expression was significantly higher in OVCAR8 (Figure 5A) and CAF primary cells (Figure 5B) compared to its expression in normal fibroblast (NF). The primary cells isolated from tissues were distinguished as CAF and NF based on the expression of a CAF specific biomarker *αSMA* which was found to be markedly increased in CAFs compared to NFs (Figure 5B).

#### 3.3.4. cpFIT-PNAs Downregulate *FLJ22447* lncRNA Expression 

**Figure 6:**
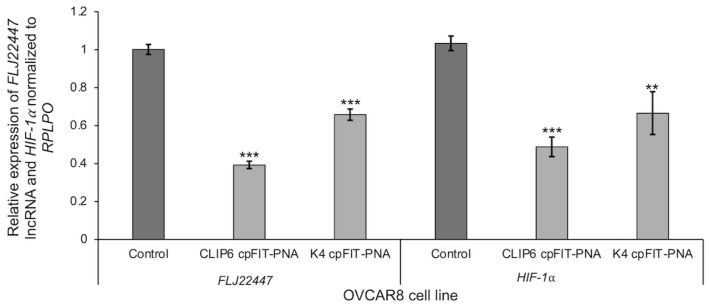
RT-qPCR analysis of *FLJ22447* lncRNA and HIF-1α expression in OVCAR8 cells treated with 2 μM K4 cpFIT-PNA and CLIP6 cpFIT-PNA for 48 h at 37 °C. cpFIT-PNA treated cells show down-regulation of *FLJ22447* lncRNA as well as reduction in HIF-1α expression. Statistical analysis performed using Student’s *t*-test; error bars represent mean ± SEM; ** *p* < 0.01, *** *p* < 0.001.

The specificity of designed K4 cpFIT-PNA and CLIP6 cpFIT-PNA towards *FLJ22447* lncRNA in OVCAR8 cells was evaluated by RT-qPCR analysis (Figure 6). OVCAR8 cells incubated with CLIP6 cpFIT-PNA and K4 cpFIT-PNA showed down regulation of FLJ22447 lncRNA expression. Moreover, the expression of *FLJ22447* lncRNA was markedly decreased in CLIP6 cpFIT-PNA in comparison to K4 cpFIT-PNA.

Further, we have checked the expression of Hypoxia-inducible factor 1-alpha gene (*HIF-1α*) in OVCAR8 cells. This oncogene is typically over-expressed in many cancers, and, therefore, may be affected indirectly by down-regulation of *FLJ22447* lncRNA. (Figure 6). As in *FLJ22447* lncRNA, down-regulation in the expression of *HIF-1α* was observed for cpFIT-PNAs in OVCAR8 cells in comparison to untreated cells. It still needs to be verified whether there is a cross-talk between these two gene targets. 

#### 3.3.5. MTT Assay to Determine the Effect of cpFIT-PNAs on Cell Viability

**Figure 7:**
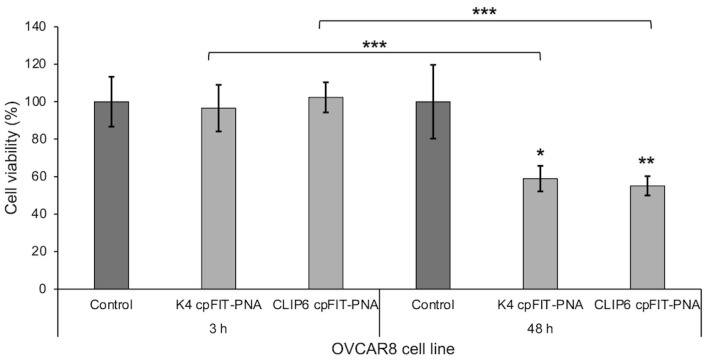
The effect of cpFIT-PNAs on the cell viability of OVCAR8 cells analyzed by MTT assay. Cells were incubated with cpFIT-PNAs (2 μM) for 3 h and 48 h. The viability of untreated OVCAR8 cells were considered as 100%. Statistical analysis performed using Student’s *t*-test; error bars represent mean ± SEM; * *p* < 0.05, ** *p* < 0.01, *** *p* < 0.001.

MTT assay was performed on OVCAR8 cells treated with K4 and CLIP6 cpFIT-PNAs (2 μM) at two different time points (3 h and 48 h). Treatment of OVCAR8 cells with both cpFIT-PNAs for 3 h did not lead to any significant reduction in cell viability. However, the percentage of viable cells after 48 h post-treatment with cpFIT-PNAs decreased considerably by 59% and 55% for K4 cpFIT-PNA and CLIP6 cpFIT-PNA, respectively. At 48 h post cpFIT-PNA treatment, both K4 cpFIT-PNA and CLIP6 cpFIT-PNA targeting *FLJ22447* lncRNA have shown considerable down-regulation in the expression of this RNA in OVCAR8 cells (Figure 7). As *FLJ22447* lncRNA is involved in the proliferation and progression of OC cells, down-regulation of *FLJ22447* lncRNA expression at 48 h post treatment in OVCAR8 cells coincides with the reduction in cell viability. Importantly, at 3 h, a timeframe suitable for diagnosis, no toxicity was noted.

## 4. Discussion

In this study, we have chosen OVCAR8 cells based on their well-known genetic similarity with patient tumor samples [58]. Initially, using synthetic RNA, we studied the photophysical properties (Figure 1) and binding affinities of FIT-PNAs and cpFIT-PNAs (Scheme 1 and Table 1) to RNA. The general observation from these studies was that the CLIP6 peptide interacts with both FIT-PNAs and cpFIT-PNAs, leading to a marked increase in duplex stabilization and to ca. 20% quenching in fluorescence. 

Simple incubation (3 h, 37 °C) of the various FIT-PNAs and cpFIT-PNAs (2 μM) in OVCAR8 cells that represent high-grade OC resulted in fluorescence to a different degree of brightness for the different FIT-PNAs (Figure 2). OVCAR8 cells express high levels of *FLJ22447* lncRNA in comparison to normal fibroblast (NF, MRC5) (Figure 5). Importantly, negligible fluorescence is shown for the scrambled sequence (K4 scr-cpFIT-PNA) (Figure 2). A direct comparison of either K4 or CLIP6 conjugated FIT-PNAs shows a notable increase in fluorescence for the cpFIT-PNAs in comparison to the unmodified FIT-PNAs. In addition, the CLIP6 CPP is superior to the simple K4 peptide. Thus, the combination of cp and CLIP6 results in a 2-fold increase in mean fluorescence in comparison to the simple design of K4 FIT-PNA.

Next, we explored the cellular uptake of FIT-PNAs (2 μM) in OVCAR8 and CAF primary cells by following flow cytometry analysis after 3 h of incubation at 37 °C (Figure 3 and Figure 4, respectively). In CAFs, lncRNAs are differentially expressed, particularly *FLJ22447*, and hence, it can be used as prognostic marker in cancer [59,60]. Since CAF provides a tumor microenvironment leading to tumor progression, migration, and invasion, using a cpFIT-PNA as an early detection of CAF specific biomarker *FLJ22447* lncRNA may provide a novel tool for the early detection of ovarian cancer. 

In OVCAR8, there is a marked difference in cellular uptake between the simple and unmodified FIT-PNA (K4 FIT-PNA) to the other FIT-PNAs and cpFIT-PNAs. Among these, the differences are not large but still appreciable with statistical significance in mean fluorescence intensity (Figure 3B). In contrast, in CAFs, the CPP (CLIP6) has a marked effect on cellular uptake (Figure 4A). Both CLIP6 FIT-PNA and CLIP6 cpFIT-PNA result in a higher mean fluorescence intensity compared to the K4 series (Figure 3B and Figure 4B). Here, the cp modification has an effect, but not as significant as the CPP (CLIP6).

Given the quenching of fluorescence observed for CLIP6 FIT-PNA (and CLIP6 cpFIT-PNA) with synthetic RNA (Figure 1), it is most reasonable to suggest that the contribution of CLIP6 to the observed diagnostic activity of cpFIT-PNA is more dominant that the introduction of the cp modifications. This contribution may be attributed to two factors: (1) higher binding affinity to RNA (Table 1), and (2) improved cellular uptake. 

We have analyzed the down-regulation of *FLJ22447* lncRNA after treating OVCAR8 cells with K4 and CLIP6 cpFIT-PNAs (2 μM) for 48 h (Figure 6). Since expression of *FLJ22447* lncRNA may be linked to hypoxia-inducible factor (*HIF-1α*) gene [5], which plays an important role in regulating the invasion and migration of ovarian cancer cells, we have also followed the expression of *HIF-1α* as a functional gene in OC cells (Figure 6). CLIP6 cpFIT-PNA is superior to K4 cpFIT-PNA, resulting in 61% and 52% down-regulation of *FLJ22447* lncRNA and *HIF-1α* levels, respectively. The differences shown in Figure 3 and Figure 4 between FIT-PNA/cpFIT-PNA uptake in OVCAR8 and CAFs may be related to the fact that CAFs are not cancer cells and, therefore, are more difficult to transfect. Thus, the CLIP6 peptide has a greater effect in CAFs. In addition, there is a difference in cellular uptake in OVCAR8 when comparing the confocal images (Figure 2) to FACS analysis (Figure 3). As these are different imaging techniques, this is not surprising. FACS is based on the analysis of thousands of cells, whereas confocal microscopy provides a snapshot of only several cells. Nonetheless, the trend is evident. That is, both cpFIT-PNA and CLIP6 improve the sensing of the FIT-PNA for this RNA biomarker.

## 5. Conclusions

In conclusion, we have demonstrated that the combination of CLIP6 and cpPNA monomers in the design of FIT-PNAs results in superior RNA sensors for the OC RNA biomarker, *FLJ22447* lncRNA. In addition, down-regulation of *FLJ22447* lncRNA may be a valid therapeutic approach. We are currently exploring other cpFIT-PNAs for various OC RNA biomarkers with the goal of establishing a panel of RNA sensors that would eventually be beneficial for detecting this disease early on.

## Figures and Tables

**Table 1:** FIT-PNA and cpFIT-PNA sequences synthesized.

Sequence	Construct	MW (Found)	MW (calc.)	Tm (°C)	ΔTm (°C)
**K4 FIT-PNA**	3′ K4-TTCTAGATT-BisQ-ACAGTTT 5′	5271.3	5276.8	50.7	
**K4 cpFIT-PNA**	3′ K4-TTCTAGAT-cpT-BisQ-cpA-CAGTTT 5′	5351.3	5356.3	54.0	3.3
**K4 scr-cpFIT-PNA**	3′ K4-TTACGGAT-cpT-BisQ-CTTATAT 5′	5309.3	5309.3		
**CLIP6 FIT-PNA**	3′ CLIP6-TTCTAGATT-BisQ-ACAGTTT 5′	6970.3	6975.84	59.3	
**CLIP6 cpFIT-PNA**	3′ CLIP6-TTCTAGAT-cpT-BisQ-cpA-CAGTTT 5′	7050.4	7053.96	62.3	3.0

Predicted and observed molecular weights of FIT-PNAs are shown. CLIP6 sequence is KVRVRVRV^D^PPTRVRERVK.

## Data Availability

All data are presented within the submitted manuscript.

## Supplementary Materials

The following supporting information can be downloaded at https://www.mdpi.com/article/10.3390/biom14060609/s1: Figures S1–S10: HPLC chromatograms and ESI-MS for all FIT-PNAs/cpFIT-PNAs; Figures S11 and S12: FACS results of duplicates for FIT-PNAs/cpFIT-PNAs in OVCAR8 and CAF cells respectively; Figures S13 and S14: Forward and sideward scatter plots (FACS) for all FIT-PNAs/cpFIT-PNAs in OVCAR8/CAF cells; Figures S15 and S16: Melting curves for FIT-PNAs/cpFIT-PNAs annealed to RNA; Tables S1 and S2: Number of total events and percentage of evaluated events in FACS experiments for FIT-PNAs in OVCAR8/CAF cells; Table S3: Primer sequences used for RT-qPCR experiments.
