# Supplementary material for: Detecting the FLJ22447 lncRNA in Ovarian Cancer with Cyclopentane-Modified FIT-PNAs (cpFIT-PNAs)
Source: Biomolecules. 2024 May 22;14(6):609. doi: 10.3390/biom14060609 (PMC11202290; doi:10.3390/biom14060609)
Supplement: Supplementary file 1 [file biomolecules-14-00609-s001.zip › biomolecules-2968788-supplementary.pdf]

Tables of contents:

HPLC and ESI-MS for K4-FIT-PNA	3
HPLC and ESI-MS for K4 scr-cpFIT-PNA	4
HPLC and ESI-MS K4 cpFIT-PNA	5
HPLC and ESI-MS CLIP6 FIT-PNA	6
HPLC and ESI-MS CLIP6 cpFLJ22447	7
FACS results in duplicates for FIT-PNAs in OVCAR8	8-9
Number of total events and percentage (FACS-OVCAR8-Table S1)	10
FACS results in duplicates for FIT-PNAs in CAFs	11-12
Number of total events and percentage (FACS-CAF-Table S2)	13
Primer sequences used for RT-qPCR experiments	13
Forward and sideward scatter plots for FACS experiments in OVCAR8	14-15
Forward and sideward scatter plots for FACS experiments in CAFs	16-17
Tm measurements	18

HPLC and ESI-MS for K4 FIT-PNA

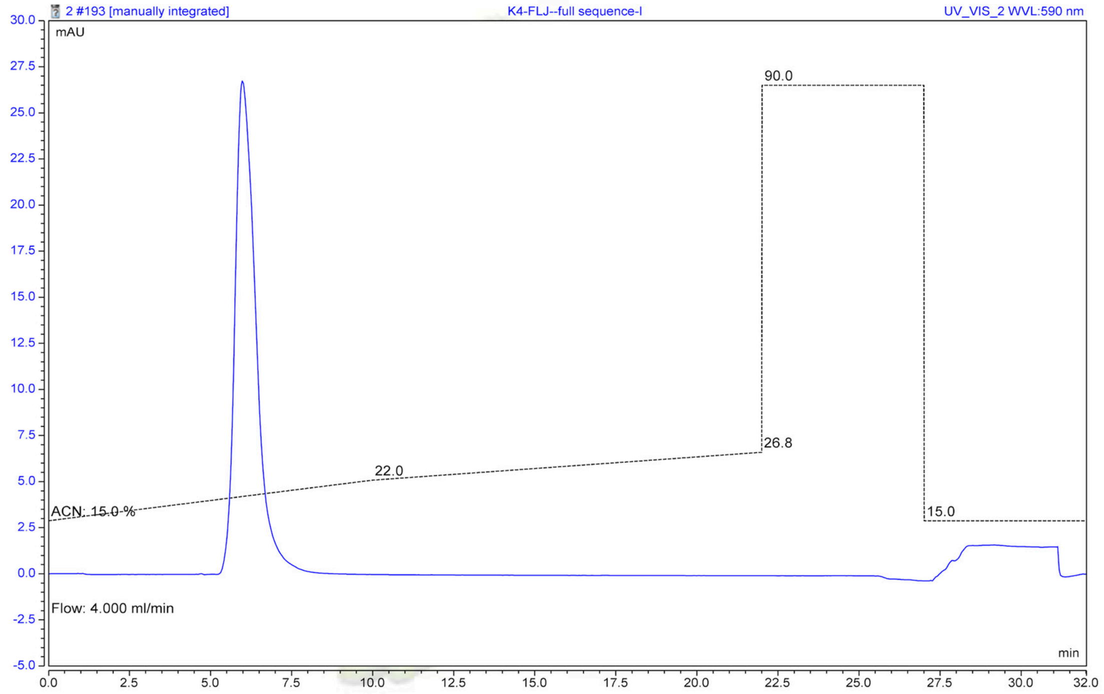

Figure S1: HPLC chromatogram of K4 FIT-PNA. Eluents: A (0.1% TFA in water) and B (ACN) were used in a linear gradient (15-22 % B in 10 min followed by 22-26.8 % B in 10 min) with a flow rate of 4 mL/min.

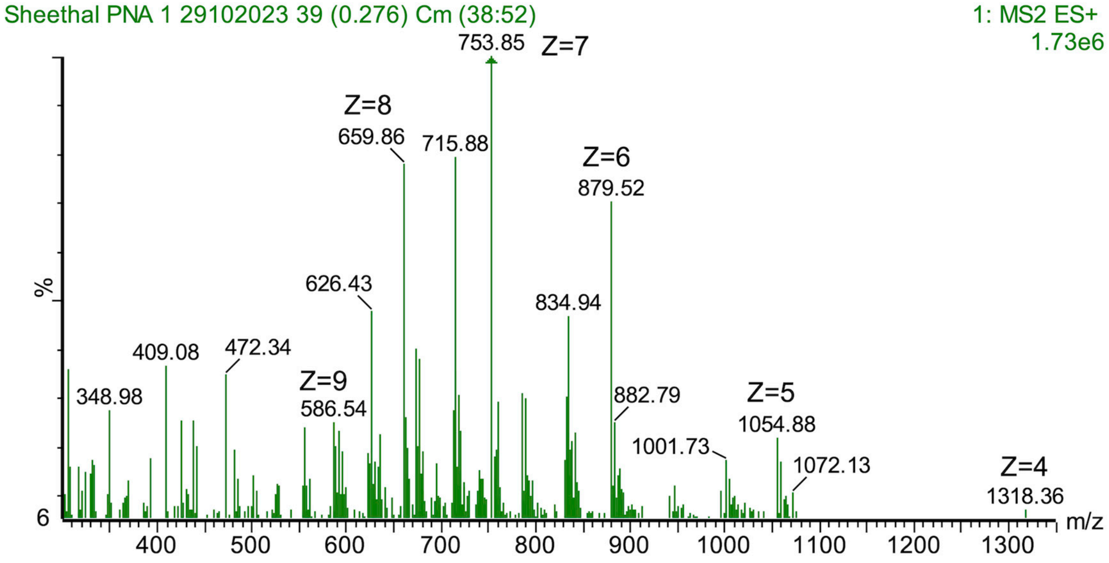

Figure S2: ESI-MS of K4 FIT-PNA. M_{calc} = 5271.3 g/mol, M_{obs} = 5276.8 g/mol.

HPLC and ESI-MS for K4 scr-cpFIT-PNA

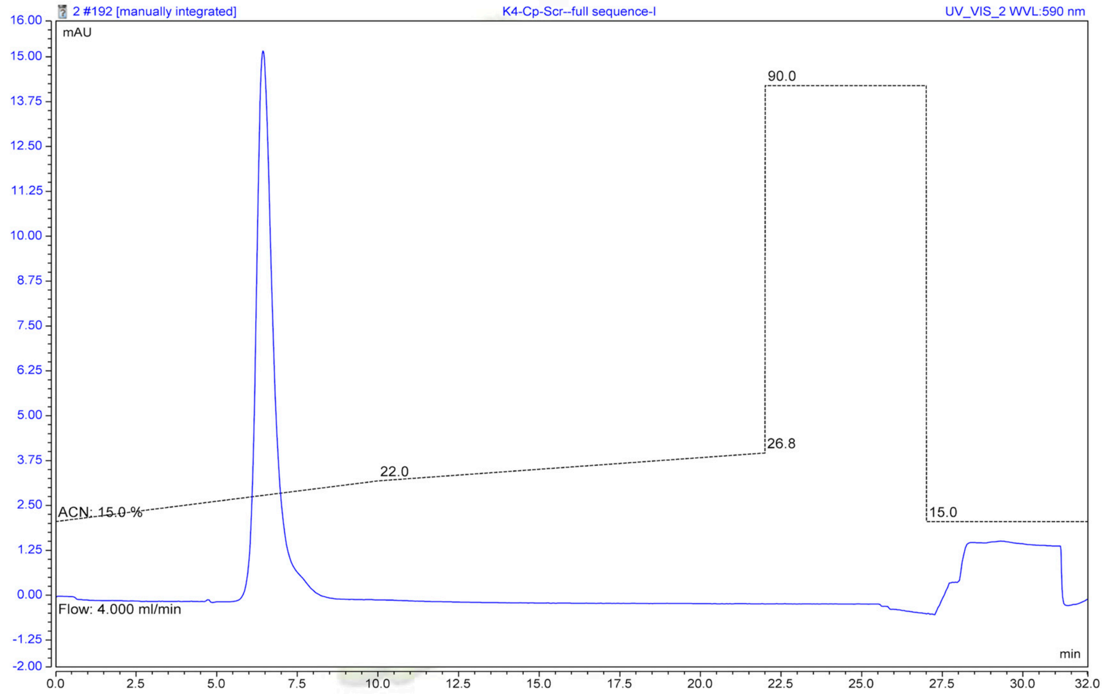

Figure S3: HPLC chromatogram of K4 scr-cpFIT-PNA. Eluents: A (0.1% TFA in water) and B (ACN) were used in a linear gradient (15-22 % B in 10 min followed by 22-26.8 % B in 10 min) with a flow rate of 4 mL/min.

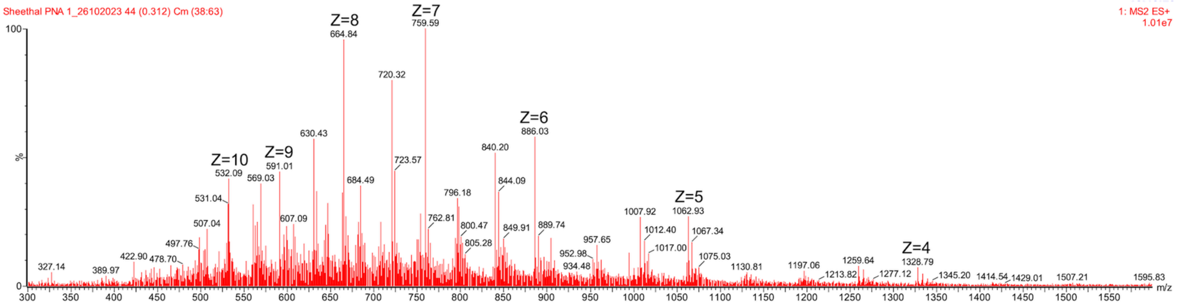

Figure S4: ESI-MS of K4 scr-cpFIT-PNA. M_{calc} = 5309.3 g/mol, M_{obs} = 5309.3 g/mol.

HPLC and ESI-MS for K4 cpFIT-PNA

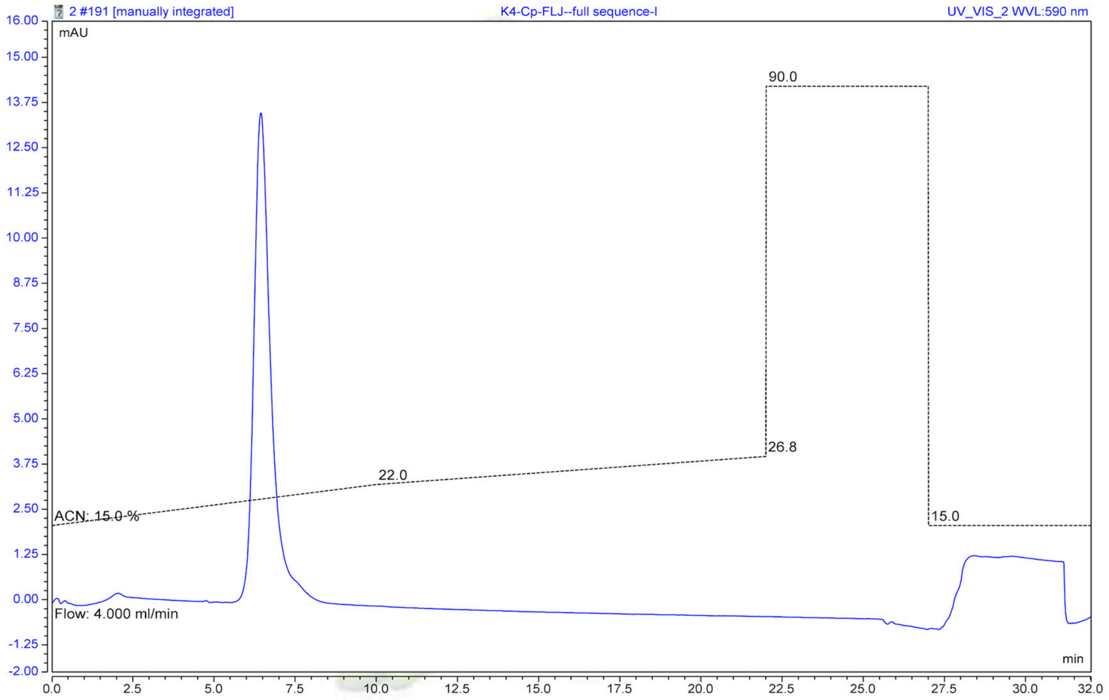

Figure S5: HPLC chromatogram of K4 cpFIT-PNA. Eluents: A (0.1% TFA in water) and B (ACN) were used in a linear gradient (15-22 % B in 10 min followed by 22-26.8 % B in 10 min) with a flow rate of 4 mL/min.

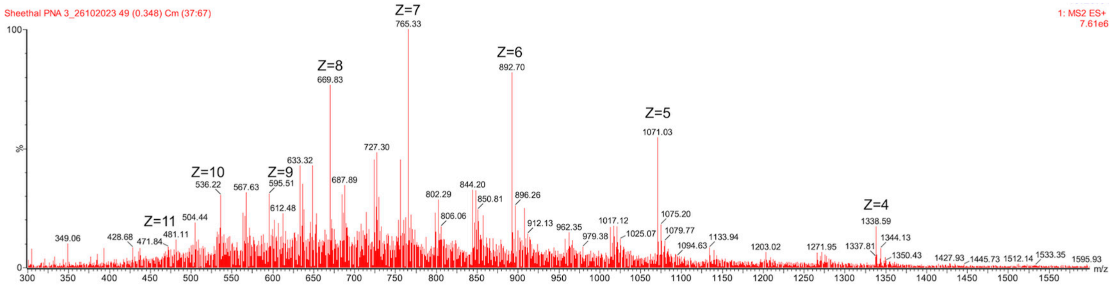

Figure S6: ESI-MS of K4 cpFIT-PNA. M_{calc} = 5351.3 g/mol, M_{obs} = 5356.3 g/mol.

HPLC and MS of CLIP6 FIT-PNA

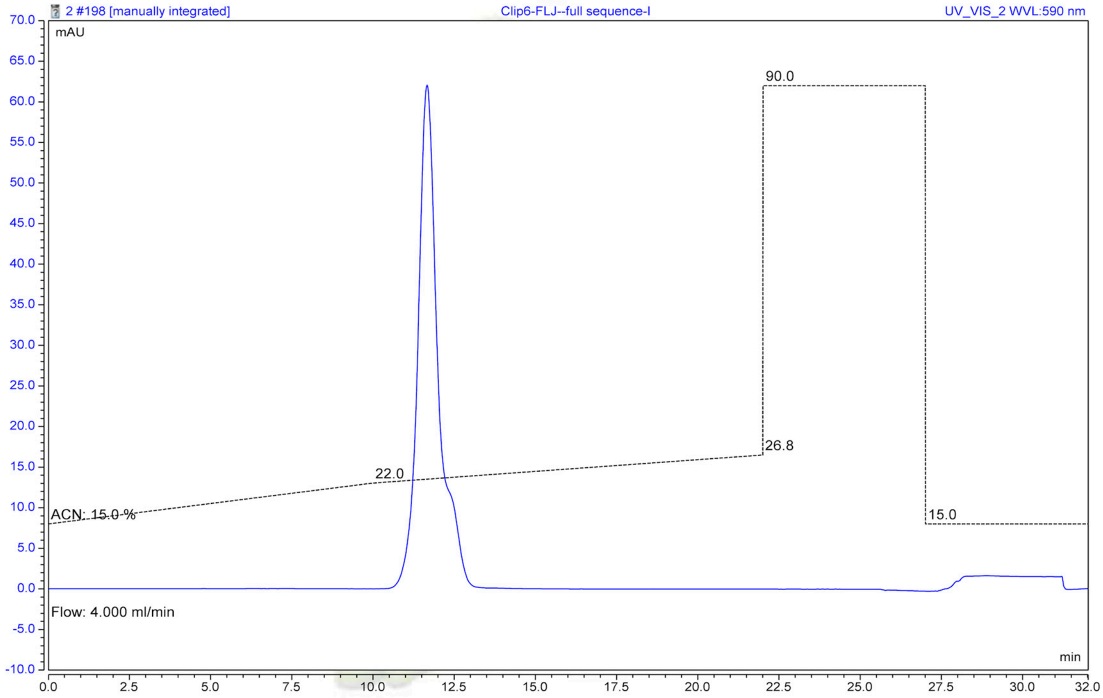

Figure S7: HPLC chromatogram of CLIP6 FIT-PNA. Eluents: A (0.1% TFA in water) and B (ACN) were used in a linear gradient (15-22 % B in 10 min followed by 22-26.8 % B in 10 min) with a flow rate of 4 mL/min.

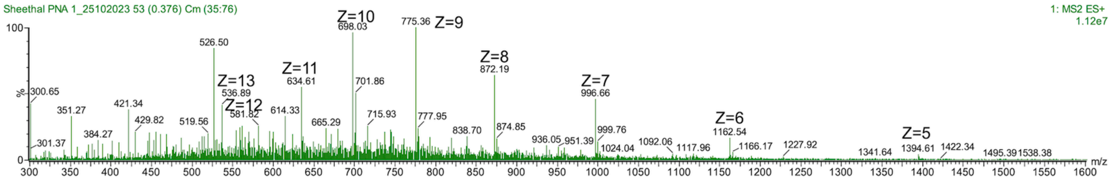

Figure S8: ESI-MS of CLIP6 FIT-PNA. M_{calc} =6970.3 g/mol, M_{obs} =6975.84 g/mol.

HPLC and MS of CLIP6 cpFIT-PNA

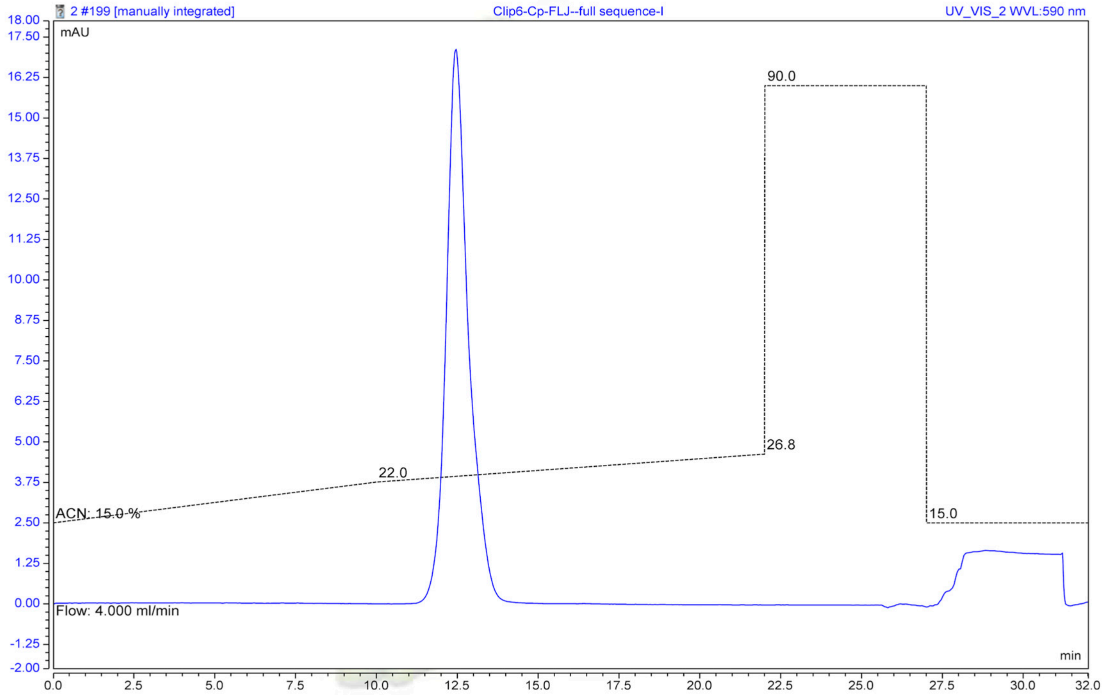

Figure S9: HPLC chromatogram of CLIP6 cpFIT-PNA. Eluents: A (0.1% TFA in water) and B (ACN) were used in a linear gradient (15-22 % B in 10 min followed by 22-26.8 % B in 10 min) with a flow rate of 4mL/min.

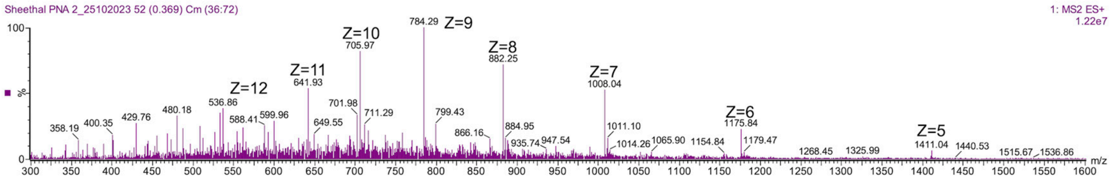

Figure S10: ESI-MS of CLIP6 cpFIT-PNA. M_{calc} = 7050.4 g/mol, M_{obs} = 7053.96 g/mol.

__FACS results of all duplicates for FIT-PNAs in OVCAR8:__ **All FACS results shown below was gated according to all positive mCherry populations.**

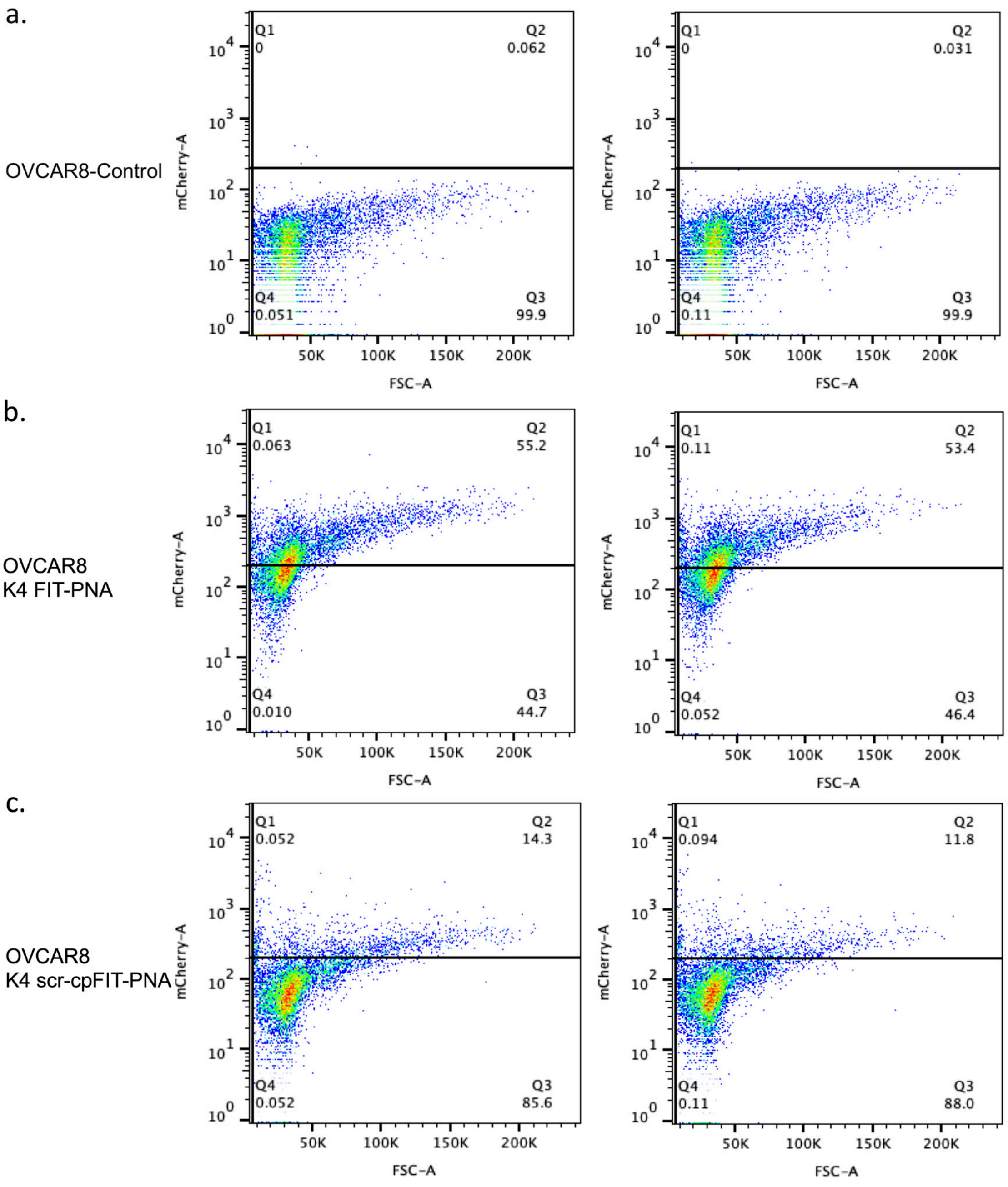

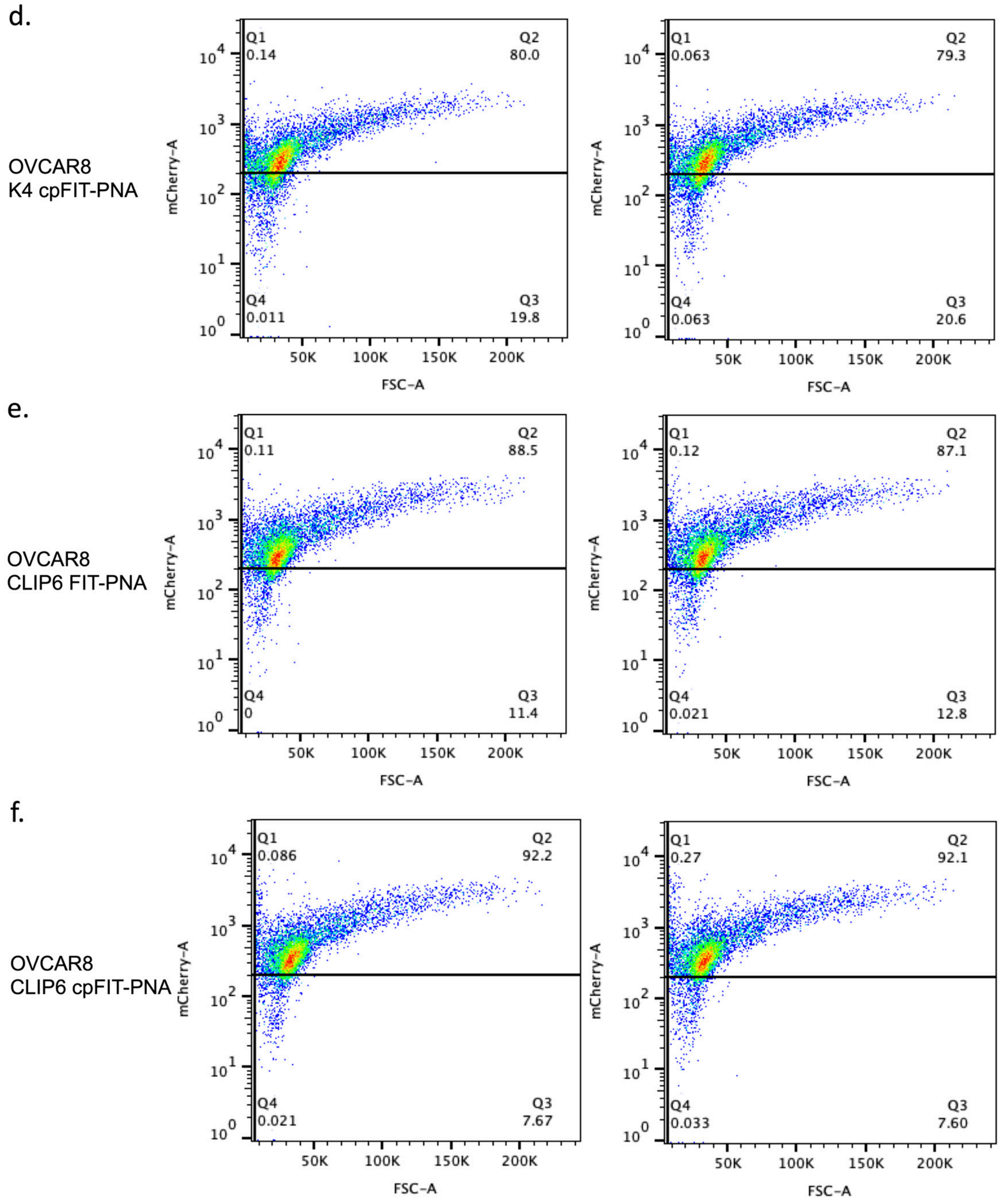

Figure S11: FACS results of duplicates for FIT-PNAs in OVCAR8 cells. OVCAR8 cells were incubated in culture media with 2 μM of FIT-PNA for 3 h, washed 3 times with 1X PBS, trypsinized and collected in 1X PBS. (a) Untreated OVCAR8 cells (served as control) (b) K4-FIT-PNA (c) K4 scr-cpFIT-PNA (d) K4 cpFIT-PNA (e) CLIP6 FIT-PNA, and (f) CLIP6 cpFIT-PNA.

OVCAR8 cells	Control		K4 FIT-PNA		K4 scr-cpFIT-PNA		K4 cpFIT-PNA		CLIP6 FIT-PNA		CLIP6 cpFIT-PNA	
**Events**	10000		10000		10000		10000		10000		10000	
**After gating on mCherry signal**	I	II	I	II	I	II	I	II	I	II	I	II
	0.06%	0.03%	55%	53%	14%	11%	80%	79%	88%	87%	92%	92%

Table S1: Number of total events and percentage of evaluated events (according to positive mCherry signal) in FACS experiments for FIT-PNAs in OVCAR8 cells.

<u>**FACS results of all duplicates for FIT-PNAs in CAFs:**</u> **All FACS results shown below were gated according to all positive mCherry populations.**

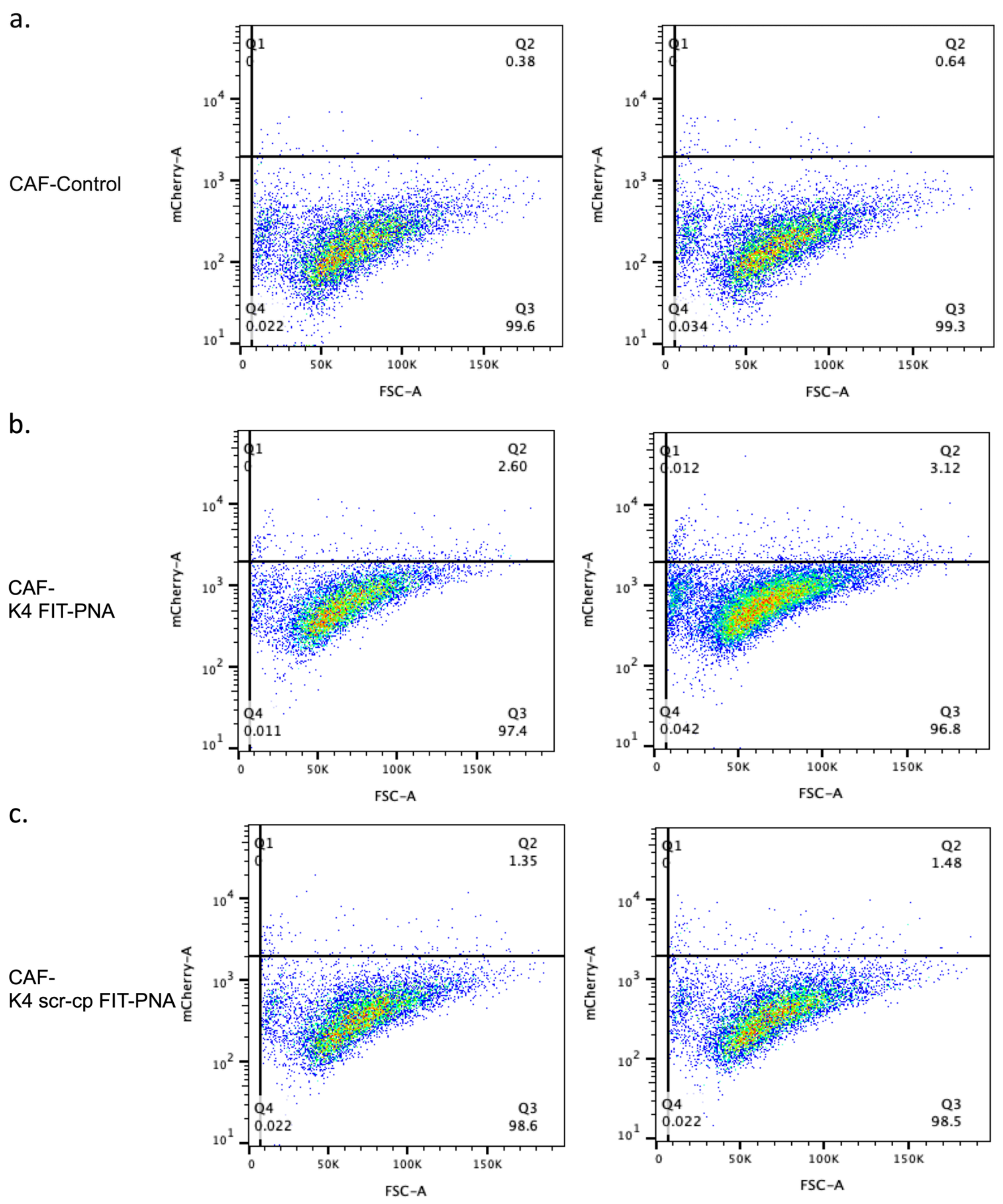

d.

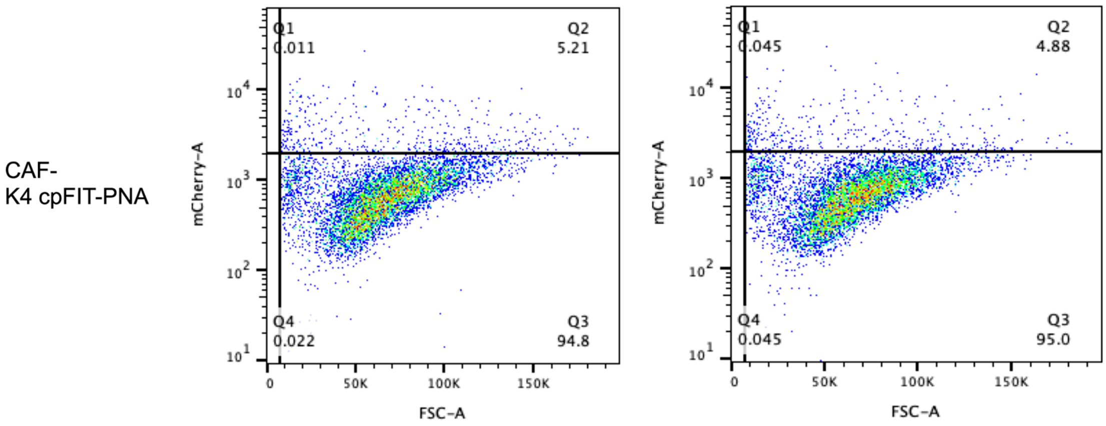

e.

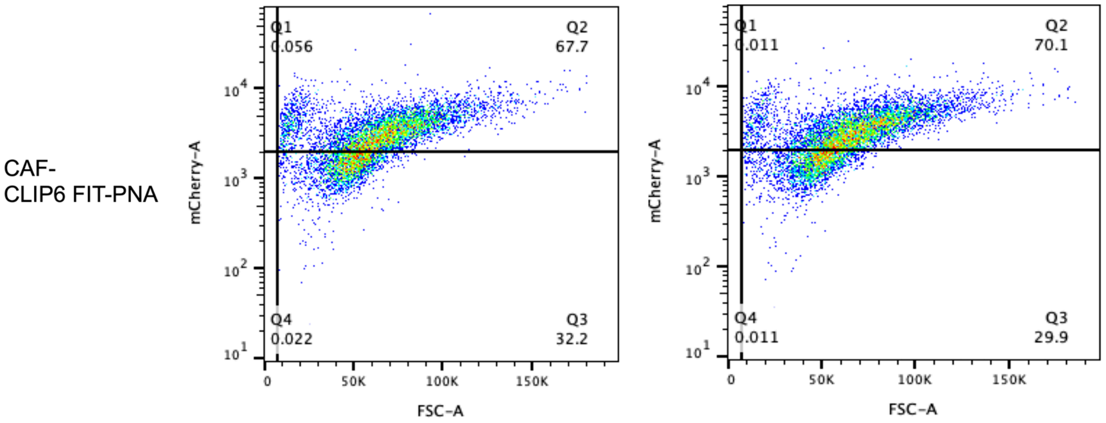

f.

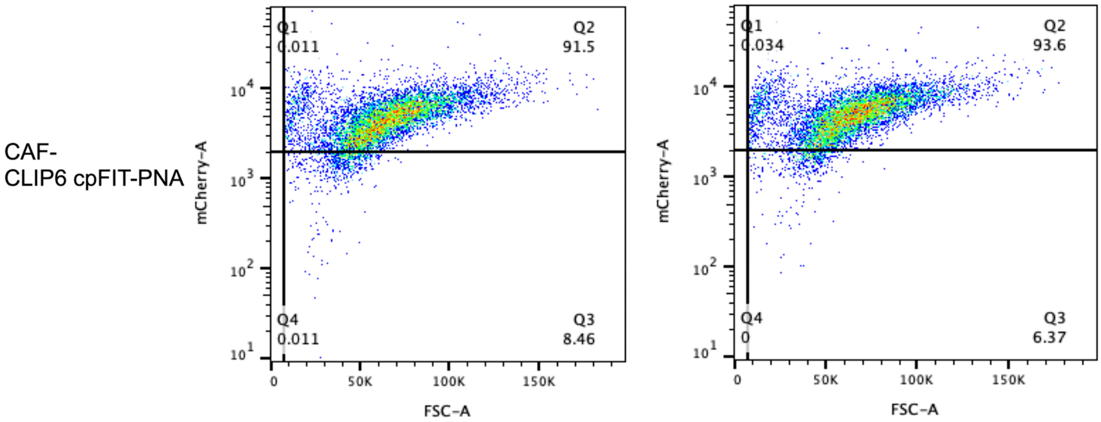

Figure S12: FACS results of duplicates for FIT-PNAs in CAF cells. CAFs were incubated in culture media with 2 μM of FIT-PNA for 3 h, washed 3 times with 1X PBS, trypsinized and collected in 1X PBS. (a) Untreated CAFs cells (as control) (b) K4 FIT-PNA (c) K4 scr-cpFIT-PNA (d) K4 cpFIT-PNA (e) CLIP6 FIT-PNA, and (f) CLIP6 cpFIT-PNA.

CAF cells	Control		K4 FIT-PNA		K4 scr-cpFIT-PNA		K4 cpFIT-PNA		CLIP6 FIT-PNA		CLIP6 cpFIT-PNA	
**Events**	10000		10000		10000		10000		10000		10000	
**After gating on mCherry signal**	I	II	I	II	I	II	I	II	I	II	I	II
	0.38%	0.64%	2.6%	3.1%	1.3%	1.4%	5.2%	4.8%	67%	70%	91%	93%

Table S2: Number of total events and percentage of evaluated events (according to positive mCherry signal) in FACS experiments for FIT-PNA in CAFs.

Primer Details

Primers	Sequence (5′-3′)
GAPDH-Forward	GACAGTCAGCCGCATCTTCT
GAPDH-Reverse	TTAAAAGCAGCCCTGGTGAC
RPLPO-Forward	CCAACTACTTCCTTAAGATCATCCAACTA
RPLPO-Reverse	ACATGCGGATCTGCTGCA
FLJ22447-Forward	TGCTTGCTCACGGGATCTTC
FLJ22447-Reverse	TTCGCATGGCTTTGACAGGT
αSMA-Forward	GCATCTGGGTGAAAGTGGT
αSMA-Reverse	GCAATGCCTCTGATTTCCAT
HIF-1α-Forward	TCCAAGAAGCCCTAACGTGT
HIF-1α-Reverse	TGATCGTCTGGCTGCTGTAA

Table S3: Primer sequences used for the RT-qPCR experiments.

Forward and sideward scatter of all FACS experiments in OVCAR8

a.

OVCAR8-Control

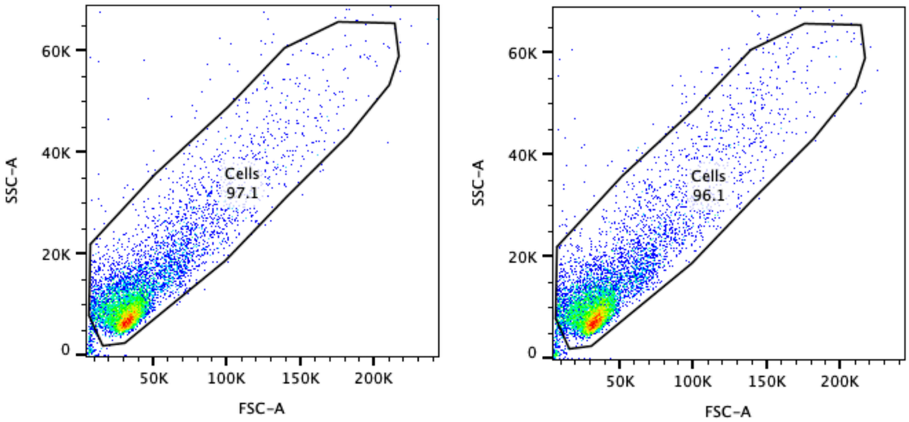

b.

OVCAR8-
K4 FIT-PNA

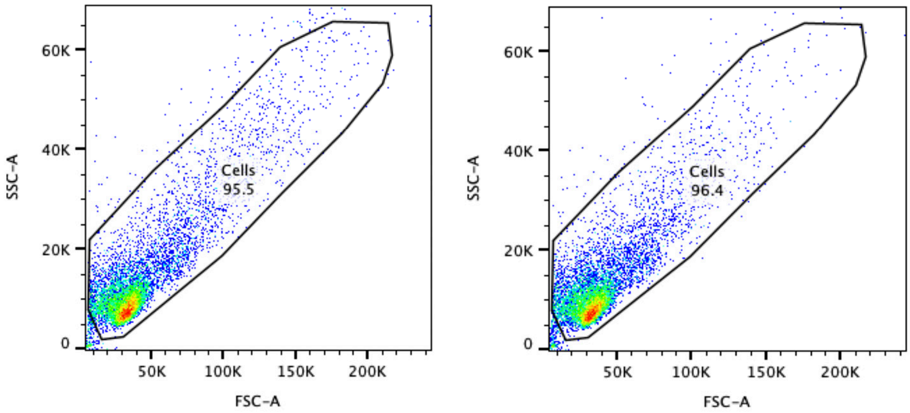

c.

OVCAR8-
K4 scr-cpFIT-PNA

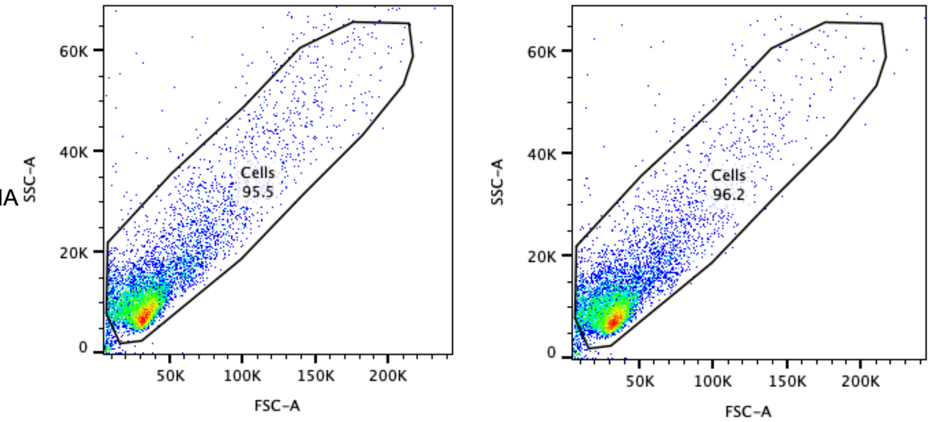

d.

OVCAR8-
K4 cpFIT-PNA

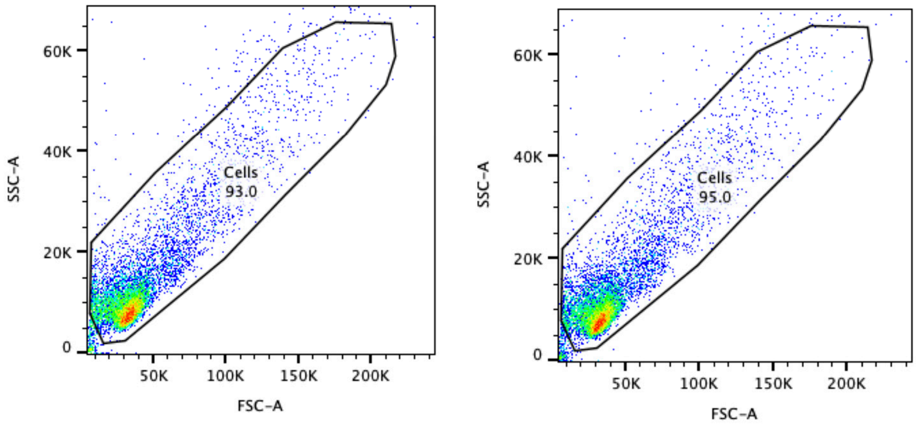

e.

OVCAR8-
CLIP6 FIT-PNA

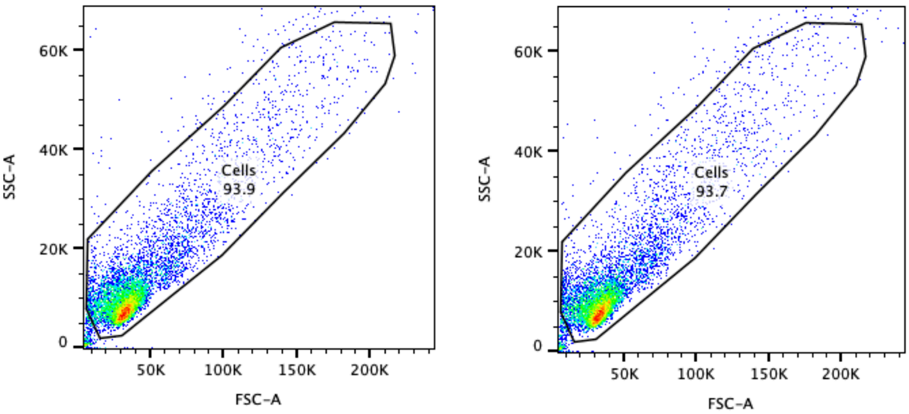

f.

OVCAR8-
CLIP6 cpFIT-PNA

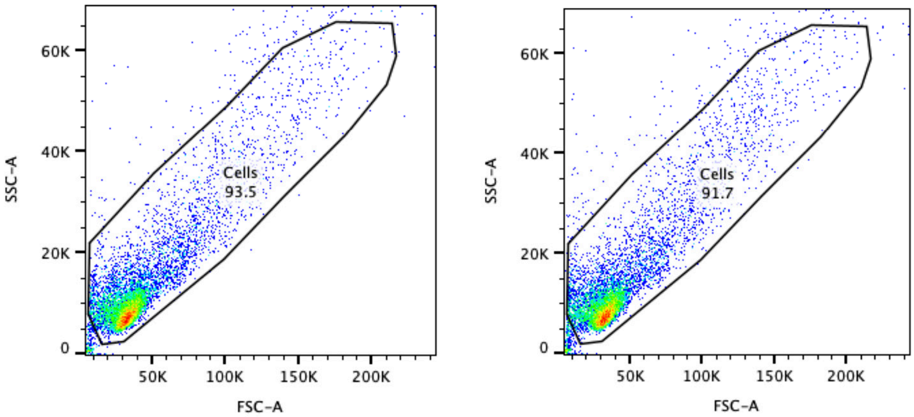

Figure S13: Forward and sideward scatter plots of all FACS experiments performed in OVCAR8.

Forward and sideward scatter of all FACS experiments in CAFs

a.

CAF-Control

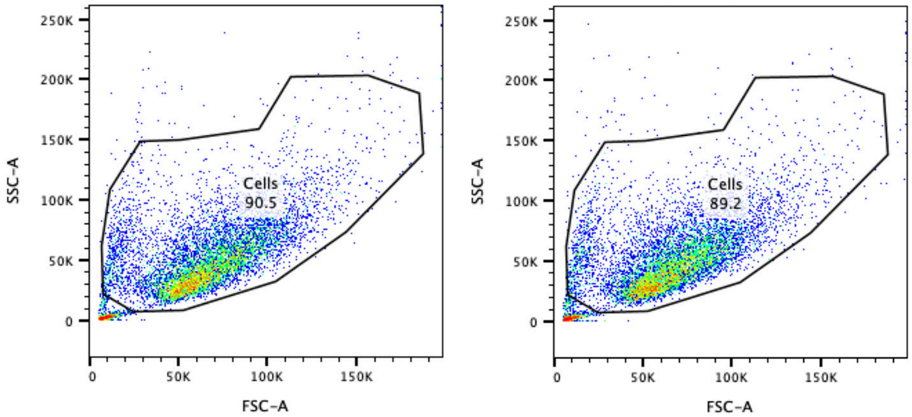

b.

CAF-
K4 FIT-PNA

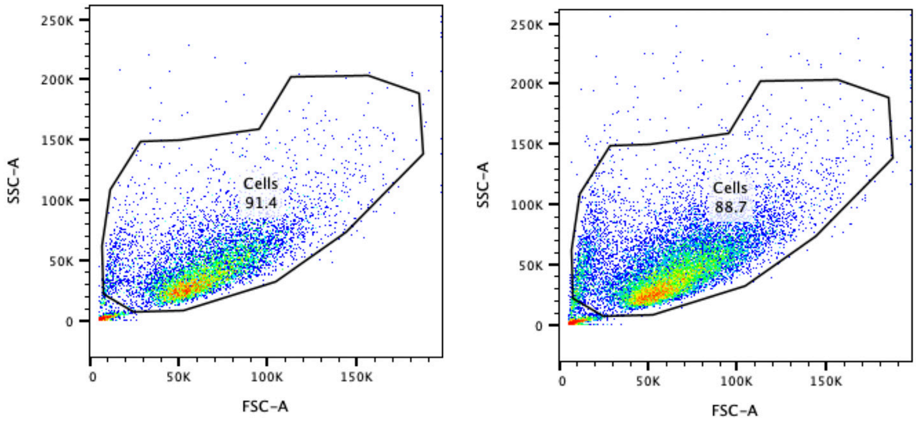

c.

CAF-
K4 scr-cpFIT-PNA

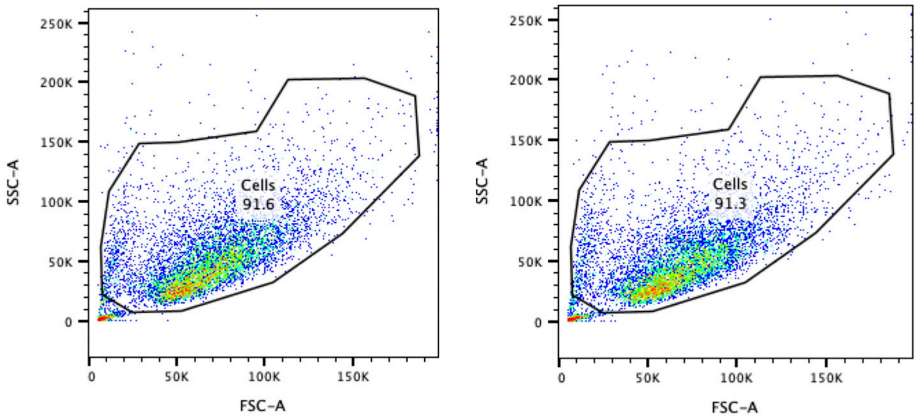

d.

CAF-
K4 cpFIT-PNA

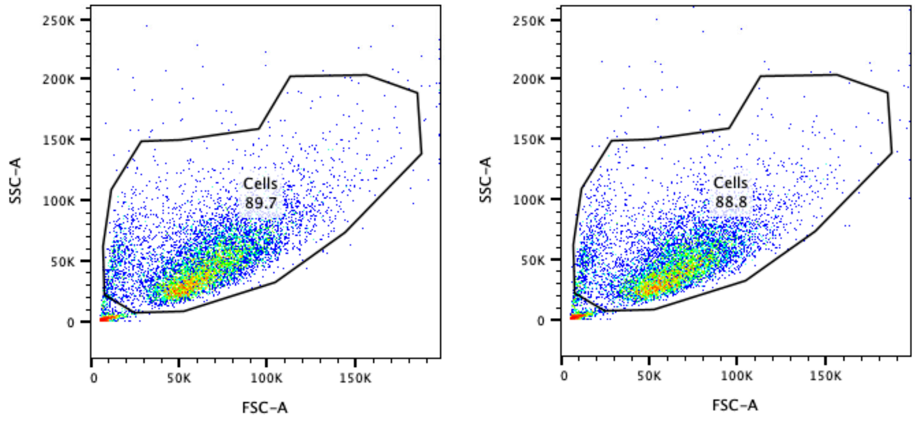

e.

CAF-
CLIP6 FIT-PNA

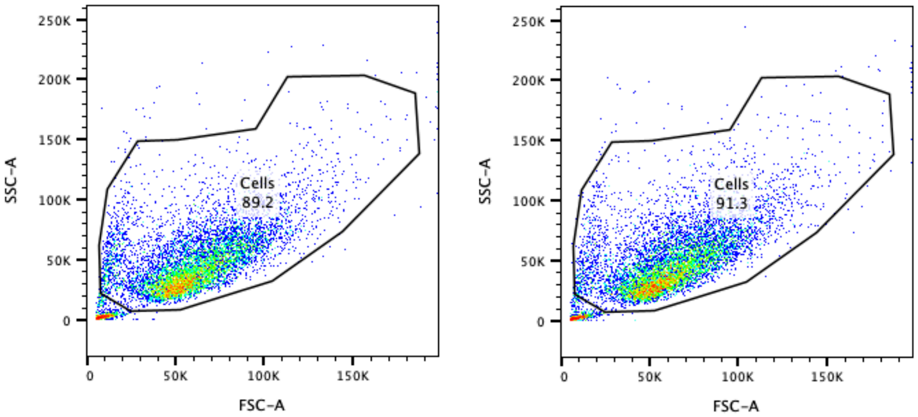

f.

CAF-
CLIP6 cpFIT-PNA

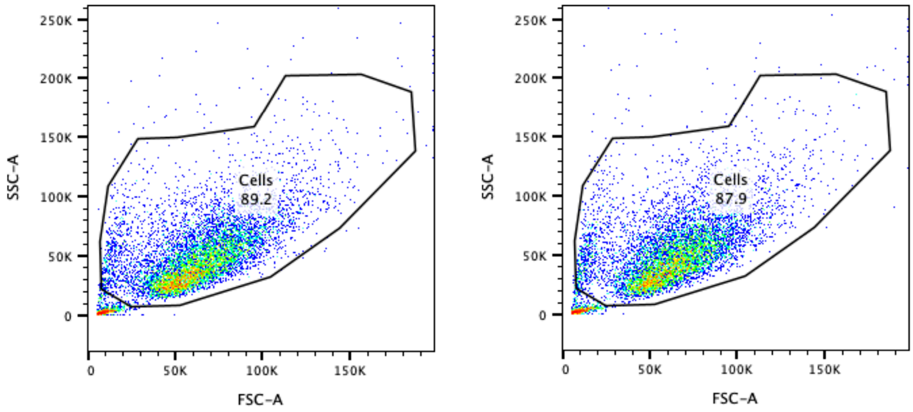

Figure S14: Forward and sideward scatter plots of all FACS experiments performed in CAF.

Tm measurements

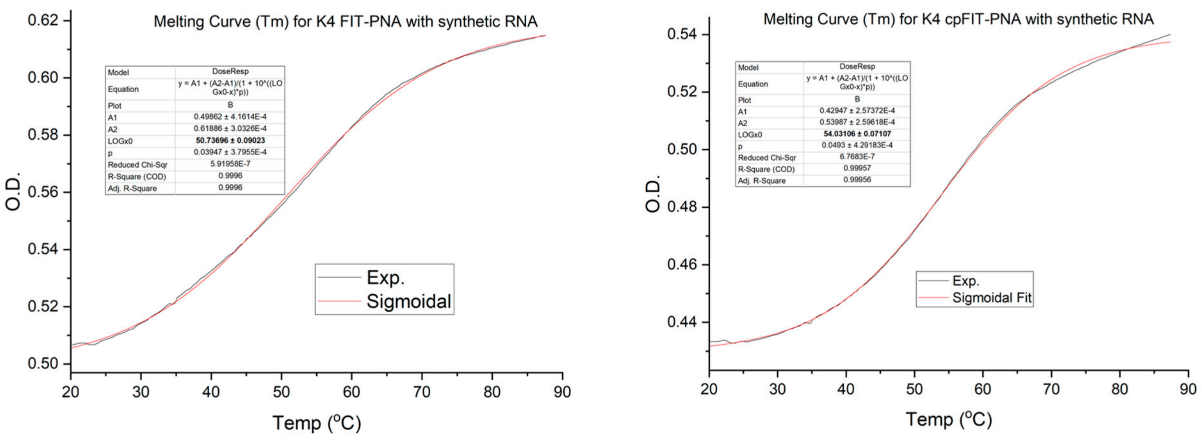

Figure S15: Melting curve profiles for K4 FIT-PNA and K4 cpFIT-PNA annealed to complementary RNA. [K4 FIT-PNA] = [K4 cpFIT-PNA] =[RNA] = 2 µM.

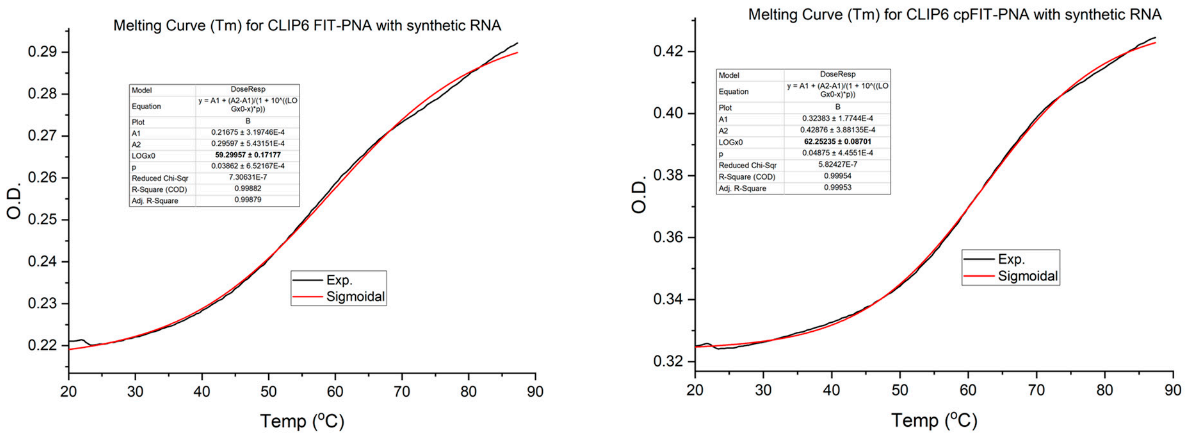

Figure S16: Melting curve profiles for K4 FIT-PNA and K4 cpFIT-PNA annealed to complementary RNA. [CLIP6 FIT-PNA] = [CLIP6 cpFIT-PNA] =[RNA] = 2 µM.
